# Supplementary material for: Metastatic prostate cancer men’s attitudes towards treatment of the local tumour and metastasis evaluative research (IP5-MATTER): protocol for a prospective, multicentre discrete choice experiment study
Source: BMJ Open. 2021 Nov 18;11(11):e048996. doi: 10.1136/bmjopen-2021-048996 (PMC8603288; doi:10.1136/bmjopen-2021-048996)
Supplement: Supplementary data [file bmjopen-2021-048996supp001.pdf]

SUPPLEMENTARY MATERIAL

1. FULL STUDY DETAILS

Full Study Title: Patients' preferences in the treatment of hormone-sensitive metastatic prostate cancer: a discrete choice experiment

Short Study title / Acronym: Metastatic prostate cancer men's Attitudes towards Treatment of the local Tumour and metastasis Evaluative Research (**IP5-MATTER**)

Product: Discrete Choice Experiment

Development Phase: Multicentre Observational Study

Sponsor: Imperial College London

Version no: 2.0

Protocol Date: 27th August 2020

**IRAS ID:**	276834
**REC Reference Number:**	20/EE/0194
**Clinical trials.gov Number:**	NCT04590976
**Sponsor Protocol Number:**	20CX5760
**Funder reference:**	Wellcome Trust. Senior Clinical Fellowship. Ref: 204998/Z/16/Z. University College Hospitals London (UCLH) Charity (P83624/1348)

2. REGULATORY, ETHICAL AND LEGAL ISSUES

2.1 Declaration of Helsinki

The investigator will ensure that this study is conducted in full conformity with the 7th revision of the 1964 Declaration of Helsinki.

2.2 Good Clinical Practice

The study will be conducted in accordance with the guidelines laid down by the International Conference on Harmonisation for Good Clinical Practice (ICH GCP E6 guidelines).

2.3 Independent Ethics Committee Approval

(i) Initial Approval

Prior to the enrolment of subjects, the REC must provide written approval of the conduct of the study at named sites, the protocol and any amendments, the Patient Information Sheet and Consent Form, any other written information that will be provided to the subjects, any advertisements that will be used and details of any subject compensation.

(ii) Approval of Amendments

Proposed amendments to the protocol and aforementioned documents must be submitted to the REC for approval as instructed by the Sponsor. Amendments requiring REC approval may be implemented only after a copy of the REC's approval letter has been obtained.

Amendments that are intended to eliminate an apparent immediate hazard to subjects may be implemented prior to receiving Sponsor or REC approval. However, in this case, approval must be obtained as soon as possible after implementation.

(iii) Annual Progress Reports

The REC will be sent annual progress reports in accordance with national requirements.

(iv) Annual Progress Reports and End of Trial Notification

The REC will be sent annual progress updates in order to facilitate their continuing review of the study (reference. ICH GCP E6 Section 3.1.4) and will also be informed about the end of the trial, within the required timelines. The Annual Progress Report will detail all SAEs recorded.

2.4 HRA approval

Health Research Authority (HRA) approval will be obtained prior to starting the study. Each participating site will confirm capacity and capability prior to commencing.
The HRA and all participating sites also need to be notified of all protocol amendments to assess whether the amendment affects the institutional approval for each site.

2.5 Other Required Approvals

None

2.6 Non-Compliance and Serious Breaches

All protocol deviations and protocol violations will be reported via MATTER trial team email address and reviewed by the Principal Investigator and reported to the trial TMG on a monthly basis. Protocol violations will be reported to the Sponsor.

An assessment of whether the protocol deviation/violation constitutes a serious breach will be made.

A serious breach is defined as:

A breach of the conditions and principles of GCP in connection with a trial or the trial protocol, which is likely to affect to a significant degree:

- The safety or physical or mental integrity of the UK trial subjects; or
- The overall scientific value of the trial

The Sponsor will be notified within 24 hours of identifying a likely Serious Breach. If a decision is made that the incident constitutes a Serious Breach, this will be reported to the REC within 7 days of becoming aware of the serious breach.

2.7 Insurance and Indemnity

Imperial College London holds negligent harm and non-negligent harm insurance policies, which apply to this study. *Imperial College London will act as the main Sponsor for this study. Delegated responsibilities will be assigned to the participating sites taking part in this study.*

2.8 Trial Registration

The study will be registered on a trial database e.g. ClinicalTrials.gov in accordance with requirements of the International Committee of Medical Journal Editors (ICMJE) regulations.

2.9 Informed Consent

Subjects will be provided with a copy of the signed Subject Information Sheet/Informed Consent Form document. The original Informed Consent Form should be retained with the source documents.

2.10 Contact with General Practitioner

It is the investigator's responsibility to inform the subject's General Practitioner (where applicable) by letter that the subject is taking part in the study provided the subject agrees to this, and information to this effect is included in the Subject Information Sheet and Informed Consent. A copy of the letter should be filed in the medical notes.

2.11 Subject Confidentiality

The investigator must ensure that the subject's confidentiality is maintained. On the CRF or other documents submitted to the Sponsors, subjects will be identified by a subject ID number only. Documents that are not submitted to the Sponsor (e.g., signed informed consent form) should be kept in a strictly confidential file by the investigator.

The investigator shall permit direct access to subjects' records and source documents for the purposes of monitoring, auditing, or inspection by the Sponsor, authorised representatives of the Sponsor and RECs.

2.12 Data Protection and Patient Confidentiality

The investigator will preserve the confidentiality of all participants taking part in the study, which will be conducted in accordance with GDPR and the Data Protection Act. Furthermore, all investigators and study staff must comply with the requirements of the Data Protection Act 2018 with regards to the collection, storage, processing and disclosure of personal information and will uphold the Act's core principles.

2.13 End of Trial

The end of the trial will be marked by the completion of the study report so that all study outcomes can be addressed. However, the trial may end early upon recommendation of the Trial Steering Committee.

2.14 Study Documentation and Data Storage

The study investigators and study site staff will comply with the requirements of the Data Protection Act 2018 concerning the collection, storage, processing and disclosure of personal information and will uphold the Act's core principles.

The investigator must retain essential documents until notified by the Sponsor, and for at least ten years after study completion. Subject files and other source data (including copies of protocols, CRFs, original reports of test results, correspondence, records of informed consent, and other documents pertaining to the conduct of the study) must be retained. Documents should be stored in such a way that they can be accessed/data retrieved at a later date.

All research documentation and information will undergo a process of pseudonymisation where possible. Whilst within the study, patients will be identified by a unique study number, and the data in the CRF will be linked to this number. Research data will be entered onto a dedicated, secure, encrypted trial database, specifically constructed for this purpose. The study team will maintain the confidentiality of all patient data and will not disclose information by which patients may be identified to any third party other than those directly involved in the treatment of the patient and organisations for which the patient has given explicit consent for data transfer. Data within the NHS system such as patient notes, and reports will remain confidential in accordance with NHS confidentiality code of practice.

Paper enrolment logs, including patients' names, NHS numbers and dates of birth, will be kept in the Investigator Site File and will be stored in a secure, locked room within each participating site. Electronic documents will be kept on secure and encrypted computers. Access to these documents will be highly restricted and will only be available to the relevant research team members.

No study documents will be destroyed without prior written agreement between the Sponsor and the investigator. Should the investigator wish to assign the study records to another party or move them to another location, written agreement must be obtained from the Sponsor.

However, psuedonymised data will be shared with Dr Verity Watson at the Health Economics Research Unit (HERU), University of Aberdeen, Aberdeen for analysis at completion of the study.

3. STUDY WITHDRAWAL

3.1 Permanent Discontinuation of Study Treatment and Withdrawal from Study

(v) Permanent discontinuation of study treatment

Subjects may discontinue study treatment for the following reasons:

- At the request of the subject.
- Adverse event/ Serious Adverse Event
- If the investigator considers that a subject's health will be compromised due to adverse events or concomitant illness that develop after entering the study.

(vi) Withdrawal from Study

Withdrawal from the study refers to discontinuation of study treatment and study procedures and can occur for the following reasons:

- Subject decision

- Loss to follow-up

3.2 Adverse Events

DEFINITIONS

Adverse Event (AE): any untoward medical occurrence in a patient or clinical study subject.

Serious Adverse Event (SAE): any untoward and unexpected medical occurrence or effect that:

- **Results in death**
- **Is life-threatening** – *refers to an event in which the subject was at risk of death at the time of the event; it does not refer to an event which hypothetically might have caused death if it were more severe*
- **Requires hospitalisation, or prolongation of existing inpatients' hospitalisation**
- **Results in persistent or significant disability or incapacity**
- **Is a congenital anomaly or birth defect**

Medical judgement should be exercised in deciding whether an AE is serious in other situations. Important AEs that are not immediately life-threatening or do not result in death or hospitalisation but may jeopardise the subject or may require intervention to prevent one of the other outcomes listed in the definition above, should also be considered serious.

REPORTING PROCEDURES

All adverse events should be reported. Depending on the nature of the event the reporting procedures below should be followed. Any questions concerning adverse event reporting should be directed to the Chief Investigator in the first instance.

Non serious AEs
All such events, whether expected or not, should be recorded.

Serious AEs
An SAE form should be completed on RedCap and also an email sent to the Chief Investigator within 24 hours. However, relapse and death due to metastatic prostate cancer, and hospitalisations for elective treatment of a pre-existing condition do not need reporting as SAEs.

All SAEs should be reported to the **HRA East of England, Cambridgeshire and Hertfordshire Research Ethics Committee (20/EE/0194)** where in the opinion of the Chief Investigator, the event was:

- 'related', ie resulted from the administration of any of the research procedures; and
- 'unexpected', ie an event that is not listed in the protocol as an expected occurrence

Reports of related and unexpected SAEs should be submitted within 15 days of the Chief Investigator becoming aware of the event, using the NRES SAE form for non-IMP studies. The Chief Investigator must also notify the Sponsor of all SAEs.

Local investigators should report any SAEs as required by their Local Research Ethics Committee, Sponsor and/or Research & Development Office

4. DATA MANAGEMENT

4.1 Source Data

Source documentation is defined as the first-time data appears, and may include original document, data and records (hospital records, clinical reports, MRI and Ultrasound reports, other procedure reports, laboratory notes, other data recorded at the pathology and biochemistry laboratories, etc.). Information in source documents (e.g. medical history) dated prior to the Informed Consent Form signature date may be used to verify patient suitability for the study.

Clinical records must be marked to indicate a subject has been enrolled into the clinical study.

The Investigator must ensure the availability of source documents from which the information on the eCRF was obtained. Where printouts and electronic medical records are provided as source documents, they should be signed and dated by a member of the adequately trained research team, to indicate that the data provided is a true reproduction of the original source document.

All study data may be inspected by the Sponsor and regulatory authorities by people working on behalf of the Sponsor, and by representatives of Regulatory Authorities, where it is relevant to this research.

4.2 Language

CRFs will be in English. Generic names for concomitant medications should be recorded in the CRF wherever possible. All written material to be used by subjects must use vocabulary that is clearly understood and be in the language appropriate for the study site.

4.3 Database

Data collection will be via Electronic Data Capture (EDC) using the online REDCap database. Data is entered into the EDC system via site personnel. All source data will be recorded in the CRF and the completed case report book will be signed by the Investigator or his/her appropriate designee. All changes made at any time following the electronic signing will have an electronic audit trail with a signature and date. The completed case report book will then again be signed by the investigator or his/her appropriate designee. Specific instructions and further details will be outlined in the CRF manual.

4.4 Data Collection

All study data will be entered into electronic Case Report (eCRFs) in a database provided by using REDCap. All eCRFs will be completed using de-identified data.

CRF completion may be delegated by the Principal Investigator (documented on the Delegated Task List) to other study personnel though the Principal Investigator remains responsible for the accuracy and integrity of all data entered to eCRFs. Further details of procedures for CRF/eCRF completion, including data review, database cleaning, issuing and resolving data queries, and identification of steps for creation, modification, maintenance and

archiving of source data via any computerised systems will be provided in the study specific Data Management Plan (CRF manual).

4.5 Archiving

All trial documentation, including that held at participating sites and the trial coordinating centre, will be archived for a minimum of 10 years following the end of the study. Subject files and other source data (including copies of protocols, CRFs, original reports of test results, correspondence, records of informed consent, and other documents pertaining to the conduct of the study) must be retained. Documents should be stored in such a way that they can be accessed/data retrieved later. Consideration should be given to security and environmental risks.

No study document will be destroyed without prior written agreement between the Sponsor and the investigator. Should the investigator wish to assign the study records to another party or move them to another location, written agreement must be obtained from the Sponsor.

Storage and handling of confidential trial data and documents will be in accordance with the Data Protection Act 2018 (UK).

5. STUDY MANAGEMENT STRUCTURE

5.1 Trial Steering Committee

A Trial Steering Committee (TSC) will be convened including as a minimum an Independent Chair, Independent Clinician, the Chief Investigator and Study Manager. The role of the TSC is to provide overall supervision of trial conduct and progress. Details of membership, responsibilities and frequency of meetings will be defined in a separate Charter.

5.2 Trial Management Group

A Trial Management Group (TMG) will be convened including the Principal Investigators, co-investigators and key collaborators, Study Statistician and Study Manager. The TMG will be responsible for day-to-day conduct of the trial and operational issues. Details of membership, responsibilities and frequency of meetings will be defined in separate Terms of Reference. Authorship of publications will be determined by the TMG. When necessary, decisions will be referred to the TSC. Meetings will be scheduled in a risk-adapted manner to allow for the review of events during the trial.

5.3 Data Monitoring Committee (DMC)

A combined data monitoring and trial steering committee will meet twice a year basis. The composition of this committee will include but not be limited to the Chief Investigator, Trial Statistician, Trial Coordinator, Research nurse and Patient representative.

5.4 Early Discontinuation of the Study

The following reasons may result in early discontinuation:

- It is not feasible to reach the planned outcomes (e.g. recruitment rate will not reach 300).

The statistical criteria for termination of the study will be detailed in the statistical analysis plan (SAP).

5.5 Risk Assessment

A study-specific risk assessment will be performed prior to the start of the study to assign a risk category of ‘low’, ‘medium’ or ‘high’ to the trial. Risk assessment will be carried out by Prof Hashim Ahmed and Mr Martin Connor in collaboration with the TMG the result will be used to guide a monitoring plan. The risk assessment will consider all aspects of the study and will be updated as required during the course of the study.

5.6 Monitoring

The study will be monitored periodically by trial monitors to assess the progress of the study, verify adherence to the protocol, ICH GCP E6 guidelines and other national/international requirements and to review the completeness, accuracy and consistency of the data.

Monitoring procedures and requirements will be documented in a Monitoring Plan, developed in accordance with the risk assessment.

5.7 Quality Control and Quality Assurance

Quality Control will be performed according to internal procedures. The study may be audited by a Quality Assurance representative of the Sponsor. All necessary data and documents will be made available for inspection.
The study may be subject to inspection and audit by regulatory bodies to ensure adherence to GCP and the UK Policy Framework for Health and Social Care Research.

5.8 Peer review

This study has been peer reviewed by The Urology Foundation (TUF), the Royal College of Surgeons of England and the British Medical Association (Appendix A). The study is funded by UCLH Charity, 5th Floor East, 250 Euston Road London, NW1 2PG (Appendix B). It has been reviewed within the Urology Department at Imperial College London and the Health Economics Research Unit, Polwarth Building, University of Aberdeen.

5.9 Patient and Public Involvement

5.10 Publication and Dissemination policy

Information concerning the study, patent applications, processes, scientific data or other pertinent information is confidential and remains the property of the Sponsor. The investigator may use this information for the purposes of the study only.

It is understood by the investigator that the Sponsor will use information developed in this clinical study in connection with the development of the ablative or radiotherapy or surgical techniques and, therefore, may disclose it as required to other clinical investigators and to Regulatory Authorities. In order to allow the use of the information derived from this clinical study, the investigator understands that he/she has an obligation to provide complete test results and all data developed during this study to the Sponsor.

Verbal or written discussion of results prior to study completion and full reporting should only be undertaken with written consent from the Sponsor.

Therefore, all information obtained as a result of the study will be regarded as CONFIDENTIAL, at least until appropriate analysis and review by the investigator(s) are completed.

Permission from the Executive/Writing Committee is necessary prior to disclosing any information relative to this study outside of the Trial Steering Committee. Any request by site investigators or other collaborators to access the study dataset must be formally reviewed by the TMG.

A Clinical Study Report summarising the study results will be prepared and submitted to the REC within a year of the end of study.

6. INFORMED CONSENT FORM

INFORMED CONSENT FORM (Phase 3/Main Study)

IP5-MATTER

Metastatic prostate cancer men's **A**ttitudes towards **T**reatment of the local **T**umour and metastasis **E**valuative **R**esearch (**IP5-MATTER**)

Chief Investigator: Professor Hashim U. Ahmed

Please initial each box below. Do not tick

1	I confirm that I have read and understand the patient information sheet dated _ _ / _ _ / _ _ _ _ (Version ___) for the **MATTER** Phase 3 / Main Study. I have had the opportunity to consider the information, ask questions and have had these answered satisfactorily.	
2	I understand that my participation is voluntary and that I am free to withdraw at any time, without giving reason and without my medical care or legal rights being affected.	
3	I understand that relevant sections of my medical notes and data collected during the study, may be looked at by individuals from the Sponsor of the trial (Imperial College London) and responsible persons authorised by the Sponsor, from regulatory authorities or from the NHS Trust, where it is relevant to my taking part in this research. I give permission for these individuals to have access to my records.	
4	I understand that I can stop the questionnaire at any time, for any reason.	
5	I understand that the information collected about me will be used to support other ethically approved research in future, and may be shared anonymously with other researchers.	
6	I give permission for the researchers to contact me regarding this trial during this trial period.	
7	I agree for my GP and other doctors to be informed of my participation in this study and of any clinical relevant study results.	
8	I agree to take part in the above study.	

All the boxes above must be initialled for consent to be valid

Site Number: INSERT
Protocol Number: 20CX5760

Version 2.0 27 AUG 2020
IRAS Number: 276834

OPTIONAL CONSENT

Please initial each box below. Do not tick

10	I give consent for information collected about me to be used to support other ethically approved research in the future, including those outside of the EEA	
11	I give consent to being contacted to potentially taking part in other ethically approved research studies.	

Name of Participant Signature Date

Name of Person taking consent Signature Date

Please give one copy of the consent form to the patient, file one copy in the patient's medical records, and retain the original in the Investigator Site File

Site Number: INSERT
Protocol Number: 20CX5760

Version 2.0 27 AUG 2020
IRAS Number: 276834
