# Supplementary material for: Metastatic prostate cancer men’s attitudes towards treatment of the local tumour and metastasis evaluative research (IP5-MATTER): protocol for a prospective, multicentre discrete choice experiment study
Source: BMJ Open. 2021 Nov 18;11(11):e048996. doi: 10.1136/bmjopen-2021-048996 (PMC8603288; doi:10.1136/bmjopen-2021-048996)
Supplement: Author's manuscript [file bmjopen-2021-048996.draft_revisions.pdf]

BMJ Open

BMJ Open is committed to open peer review. As part of this commitment we make the peer review history of every article we publish publicly available.

When an article is published we post the peer reviewers' comments and the authors' responses online. We also post the versions of the paper that were used during peer review. These are the versions that the peer review comments apply to.

The versions of the paper that follow are the versions that were submitted during the peer review process. They are not the versions of record or the final published versions. They should not be cited or distributed as the published version of this manuscript.

BMJ Open is an open access journal and the full, final, typeset and author-corrected version of record of the manuscript is available on our site with no access controls, subscription charges or pay-per-view fees (http://bmjopen.bmj.com).

If you have any questions on BMJ Open's open peer review process please email
info.bmjopen@bmj.com

BMJ Open

Metastatic prostate cancer men's Attitudes towards Treatment of the local Tumour and metastasis Evaluative Research (IP5-MATTER): Protocol for a prospective, multicentre discrete choice experiment study

Journal:	*BMJ Open*
Manuscript ID	bmjopen-2021-048996
Article Type:	Protocol
Date Submitted by the Author:	14-Jan-2021
Complete List of Authors:	Connor, Martin; Imperial College London, Department of Surgery and Cancer<br>Genie, Mesfin G; University of Aberdeen, Health Economics Research Unit; Ca' Foscari University of Venice, Economics<br>Gonzalez, Michael; Imperial College Healthcare NHS Trust, Department of Oncology<br>Sarwar, Naveed; Imperial College Healthcare NHS Trust, Department of Oncology<br>Thippu Jayaprakash, Kamalram; Addenbrooke's Hospital, Oncology; Queen Elizabeth Hospital King's Lynn NHS Foundation Trust, Oncology<br>Horan, Gail; Addenbrooke's Hospital, Department of Oncology<br>Hosking-Jervis, Feargus; Imperial College London Division of Surgery, Imperial College London<br>Klimowska-Nassar, Natalia; Imperial College London, Department of Surgery and Cancer; Imperial College London, Imperial College Clinical Trials Unit<br>Sukumar, Johanna; Imperial College London, Department of Surgery and Cancer; Imperial College London, Imperial College Clinical Trials Unit<br>Pokrovska, Tzveta; Imperial College Healthcare NHS Trust, Department of Oncology<br>Basak, Dolan; Imperial College Healthcare NHS Trust, Department of Oncology<br>Robinson , Angus ; Brighton and Sussex University Hospitals NHS Trust, Department of Oncology<br>Beresford, Mark; Royal United Hospitals Bath NHS Foundation Trust, Department of Oncology<br>Rai, Bhavan; Newcastle Upon Tyne Hospitals NHS Foundation Trust, Department of Urology<br>Mangar, Stephen; Imperial College Healthcare NHS Trust, Department of Oncology<br>Khoo, Vincent; Royal Marsden NHS Foundation Trust, Department of Oncology<br>Dudderidge, Tim; University Hospital Southampton NHS Foundation Trust, Department of Urology<br>Falconer, Alison; Imperial College Healthcare NHS Trust, Department of Oncology<br>Winkler, Mathias; Imperial College London, Department of Surgery and Cancer; Imperial College Healthcare NHS Trust, Imperial Urology

Watson, Verity; University of Aberdeen, Health Economics Research Unit (HERU)
Ahmed, Hashim; Imperial College London Division of Surgery, Imperial College London

Keywords: Prostate disease < UROLOGY, HEALTH ECONOMICS, Urological tumours < ONCOLOGY, RADIOTHERAPY, SURGERY, Radiation oncology < RADIOLOGY & IMAGING

For peer review only - http://bmjopen.bmj.com/site/about/guidelines.xhtml

I, the Submitting Author has the right to grant and does grant on behalf of all authors of the Work (as defined in the below author licence), an exclusive licence and/or a non-exclusive licence for contributions from authors who are: i) UK Crown employees; ii) where BMJ has agreed a CC-BY licence shall apply, and/or iii) in accordance with the terms applicable for US Federal Government officers or employees acting as part of their official duties; on a worldwide, perpetual, irrevocable, royalty-free basis to BMJ Publishing Group Ltd ("BMJ") its licensees and where the relevant Journal is co-owned by BMJ to the co-owners of the Journal, to publish the Work in this journal and any other BMJ products and to exploit all rights, as set out in our licence.

The Submitting Author accepts and understands that any supply made under these terms is made by BMJ to the Submitting Author unless you are acting as an employee on behalf of your employer or a postgraduate student of an affiliated institution which is paying any applicable article publishing charge ("APC") for Open Access articles. Where the Submitting Author wishes to make the Work available on an Open Access basis (and intends to pay the relevant APC), the terms of reuse of such Open Access shall be governed by a Creative Commons licence – details of these licences and which Creative Commons licence will apply to this Work are set out in our licence referred to above.

Other than as permitted in any relevant BMJ Author's Self Archiving Policies, I confirm this Work has not been accepted for publication elsewhere, is not being considered for publication elsewhere and does not duplicate material already published. I confirm all authors consent to publication of this Work and authorise the granting of this licence.

For peer review only

Metastatic prostate cancer men's Attitudes towards Treatment of the local Tumour and metastasis Evaluative Research (IP5-MATTER): Protocol for a prospective, multicentre discrete choice experiment study

Martin J. Connor [1,2,3], Mesfin G. Genie [4], Michael Gonzalez [5], Naveed Sarwar [5], Kamalram Thippu Jayaprakash [6], Gail Horan [6], Feargus Hosking-Jervis [1], Natalia Klimowska-Nassar [1,7], Johanna Sukumar [1,7], Tzveta Pokrovska [5], Dolan Basak [5], Angus Robinson [8], Mark Beresford [9], Bhavan Rai [10], Stephen Mangar [5], Vincent Khoo [11], Tim Dudderidge [12], Alison Falconer [5], Mathias Winkler [1,2,3], Verity Watson [4], Hashim U. Ahmed [1,2]

1. Imperial Prostate, Division of Surgery, Department of Surgery and Cancer, Faculty of Medicine, Imperial College London, London, UB1 3HW
2. Imperial Urology, Imperial College Healthcare NHS Trust, London, W6 8RF
3. Department of Urology, West Middlesex University Hospital (WMUH), Chelsea & Westminster NHS Foundation Trust, Isleworth, Middlesex, TW7 6AF
4. Health Economic Research Unit (HERU), Faculty of Medicine, University of Aberdeen, Aberdeen
5. Department of Oncology, Imperial College Healthcare NHS Foundation Trust, Charing Cross Hospital, London, W6 8RF
6. Department of Oncology, Addenbrooke's Hospital and The Queen Elizabeth Hospital Kings Lynn NHS Foundation, Norfolk, PE30 4ET
7. Imperial Clinical Trials Unit (ICTU), School of Public Health, Faculty of Medicine, Imperial College London, London, UB1 3HW
8. Department of Oncology, Brighton and Sussex University Hospitals NHS Trust, Brighton BN2 1ES
9. Department of Oncology, The Royal United Hospitals Bath, Bath, BA1 3NG
10. Department of Urology, Northern Centre for Cancer Care, Newcastle upon Tyne Hospitals NHS Foundation Trust, NE7 7DN
11. Department of Clinical Oncology, The Royal Marsden Hospital & Institute of Cancer Research, London, SW3 6JJ

For peer review only - http://bmjopen.bmj.com/site/about/guidelines.xhtml

12. Department of Urology, University Hospital Southampton NHS Foundation Trust, Southampton, SO16 6YD

Corresponding Author:
Mr. Martin J. Connor
Division of Surgery
Department of Surgery & Cancer
Imperial College London
Room 5L.28
Charing Cross Lab Block
martin.connor2@nhs.net

Main text: 2,791 Words

Keywords: *prostate cancer, metastatic, radiotherapy, radical prostatectomy, cytoreductive, SABR, SBRT, stereotactic radiotherapy, high intensity focussed ultrasound, cryotherapy, oligometastatic, choice behaviour, health economics*

For peer review only - http://bmjopen.bmj.com/site/about/guidelines.xhtml

Abstract

Introduction: Systemic therapy with androgen deprivation therapy (ADT) and intensification with agents such as Docetaxel, Abiraterone Acetate and Enzalutamide, has resulted in improved overall survival in men with *de novo* synchronous metastatic hormone-sensitive prostate cancer (mHSPC). Novel local cytoreductive treatments and metastasis-directed therapy are now being evaluated. Such interventions may provide added survival benefit or delay the requirement for further systemic agents and associated toxicity but can confer additional harm. Understanding men's preferences for treatment options in this disease state is crucial for patients, clinicians, carers and future healthcare service providers.

Methods: Using a prospective, multi-centre discrete choice experiment (DCE), we aim to determine the attributes associated with treatment that are most important to men with mHSPC. Furthermore, we plan to determine men's preferences for, and trade-offs between, the attributes (survival and side-effects) of different treatment options including systemic therapy, local cytoreductive approaches (external beam radiotherapy, cytoreductive radical prostatectomy or minimally-invasive ablative therapy) and metastases-directed therapies (metastasectomy or stereotactic ablative body radiotherapy). All men with newly-diagnosed mHSPC within four months of commencing ADT and WHO performance status 0 to 2 are eligible. Men who have previously consented to a cytoreductive treatment or have developed castrate-resistant disease will be excluded. This study includes a qualitative analysis component, with patients (*n*=15) and healthcare professionals (*n*=5), to identify and define the key attributes associated with treatment options that would warrant trade-off evaluation in a DCE. The main phase component planned recruitment is 300 patients over one year, commencing in January 2021, with planned study completion in March 2022.

Ethics and dissemination: Approved by Health Research Authority East of England, Cambridgeshire and Hertfordshire Research Ethics Committee (20/EE/0194). Funded by the Wellcome Trust (204998/Z/16/Z) & University College London Hospitals (UCLH) Charity (P83624/1348). Results will be published in peer-reviewed journals.

Trial Registration details: NCT04590976

For peer review only - http://bmjopen.bmj.com/site/about/guidelines.xhtml

STRENGTHS AND LIMITATIONS OF THIS STUDY

1. IP5-MATTER is the first discrete choice experiment evaluating men's treatment preferences for local cytoreductive and metastasis-directed therapy in mHSPC.
2. This study will determine men's preferences for, and trade-offs between, the attributes (survival and side-effects) posed by different treatment options in metastatic prostate cancer that are considered in the decision-making process at disease presentation.
3. The study will be used to determine if, on average, men are willing to accept the potential effect sizes that are reported in randomised studies.
4. The current evidence-base for local cytoreductive and metastasis-directed interventions continues to evolve, enabling a broad range of hypothetical risks and benefits to be considered.
5. Recruitment for this study will occur within the United Kingdom, which may limit the generalisability of findings to other men with mHSPC populations.

For peer review only - http://bmjopen.bmj.com/site/about/guidelines.xhtml

INTRODUCTION

The number of men diagnosed with prostate cancer each year in the United Kingdom (UK) is reported to be 47,000 [1]. In this cohort it is estimated that, 4,500 men will have a diagnosis of *de novo* synchronous metastatic hormone-sensitive prostate cancer (mHSPC) at index presentation [1]. The mainstay of treatment for such men has been hormonal castration with androgen deprivation therapy (ADT) alone. In isolation this treatment modality results in the emergence of a castrate-resistant state within a median of 11 to 18 months that restricts overall survival (OS) to 3.5 years [2, 3].

Recent advances with using ADT in combination with Docetaxel or novel anti-androgens (e.g. Enzalutamide, Abiraterone Acetate, Apalutamide) at diagnosis have led to a rise in the median OS to 4.8 years [3-9]. Current research is considering the role of local cytoreductive approaches (cytoreductive radical prostatectomy, external beam radiotherapy or minimally-invasive ablative therapy) and metastasis-directed interventions (predominantly, surgical metastatectomy or stereotactic ablative body radiotherapy [SABR]) with a view to acquiring further oncological benefit in this setting [1, 10-18].

Research into the role of local cytoreductive and metastasis-directed interventions has, in part, been propagated by the emergence of the principle of an ‘oligo-metastatic’ state [19]. In this context, men are hypothesised to harbour a favourable clinically-defined, low disease burden that lies between ‘locally-advanced’ and ‘poly-metastatic’ disease [19-21]. Early randomised data suggests that men who exhibit such disease may gain the most benefit from additional cytoreductive interventions [10, 22].

These interventions offer potential overall and progression-free survival benefits. Furthermore, they may allow second-line systemic agents and their associated toxicities to be avoided or deferred. However, we currently understand very little about men’s treatment preferences and the decision-making process in this emerging treatment paradigm.

The IP5-MATTER study will use a Discrete Choice Experiment (DCE) to determine men’s preferences for, and trade-offs between, the attributes (survival and side-

For peer review only

For peer review only - http://bmjopen.bmj.com/site/about/guidelines.xhtml

effects) of different treatment options for mHSPC including systemic therapy, local cytoreductive and metastases-directed therapies.

DISCRETE CHOICE EXPERIMENT (DCE)

A DCE is a survey method that is related to ‘conjoint analysis’ [23, 24]. A DCE was selected as the method for this study as it allows the researcher to calculate the trade-offs that patients make between specific treatment options [25]. The trade-off in IP5-MATTER is whether a man is willing to accept the disadvantages to himself (i.e. side effects, risk, visits to hospitals) with an extra treatment(s) in exchange for potential survival or cancer progression-free survival benefits. This is also termed the “marginal rate of substitution” [25].

In the IP5-MATTER study, patients will be presented with hypothetical treatment scenarios [25]. Each scenario will be described using a set of ‘attributes’ associated with each treatment e.g. treatment modality, urine incontinence, fatigue, loss of erections, length of time until cancer starts to grow again, length of survival after diagnosis. Each treatment scenario will be associated with a realisation or level for each attribute. For instance, in a hypothetical treatment the urine incontinence attribute may take the value of 10% or 1 in 10 men, i.e. of 100 men who have the hypothetical treatment 1 in 10 will experience urine incontinence.

The attributes and levels will be combined into hypothetical treatment scenarios and these will be grouped into treatment choice sets using experimental design theory. In essence, the patient will be asked to ‘choose’ between two treatments A or B in a choice set.

Each patient will be asked to complete several choice sets each containing different pairs of treatments. Each choice set will vary the levels of the attributes in a systematic way that allows the researcher to identify how the different realisations of the attributes affects the patients’ choices (**Figure 1**). We will include up to 12 choices per patient [23, 24].

For peer review only

PREVIOUS DISCRETE CHOICE EXPERIEMENTS IN PROSTATE CANCER

A number of DCE studies have previously been completed in the field of non-metastatic prostate cancer, metastatic castrate-resistant prostate cancer (mCRPC) and mHSPC suitable to elicit patients' treatment preferences [26-30].

Non-Metastatic Prostate Cancer

Sculpher et al. undertook the first prostate cancer DCE in 129 men in the UK with non-metastatic prostate cancer (T1 or T2) [30]. The primary focus was to evaluate side-effects of various ADT options. The group reported men were willing to forgo up to 3 months of life expectancy to avoid limitations in physical energy [30].

The recent COMPARE (COMparing treatment options for ProstAte cancer) multicentre study focused on 650 men with newly-diagnosed localised non-metastatic prostate cancer [NCT01177865] [31]. Watson and colleagues identified that patients' preferences for attributes for treatment were, in order: survival, no incontinence, not needing further treatment and maintaining an erection. Interestingly, men were willing to trade 0.68% absolute survival for a 1% absolute improvement in urinary function and 0.28% absolute survival for 1% absolute improvement in maintaining erections [31].

Furthermore, King and colleagues in a study of 357 men with localised prostate cancer, who had undergone either radical therapy or commenced ADT, found men were similarly willing to trade-off 6 months of survival if they could avoid erectile dysfunction and approximately 13 months of survival if they could avoid urinary incontinence [27].

Finally, in a study of 110 men with early prostate cancer, as well as their treating urologist, conducted by de Bekker-Grob and colleagues it was reported that whilst urinary incontinence was a concern among both patient and clinician, the risk of erectile dysfunction secondary to radiotherapy was solely valued by the clinician alone [26].

For peer review only - http://bmjopen.bmj.com/site/about/guidelines.xhtml

Metastatic Castrate-Resistant Prostate Cancer

In the mCRPC setting, Eliasson and colleagues study 285 men with mCRPC from three countries (Germany, France and the United Kingdom) [29]. The group reported that lower fatigue (Odds Ratio, 1.365 [95% CI 1.219-1.528]), and fewer additional hospital visits (Odds Ratio, 1.245 [95%CI 1.111-1.397]) were the most important factors affecting any treatment choice [29].

Furthermore, Uemura and colleagues studied 133 Japanese men with mCRPC to demonstrate that fatigue (relative importance [RI] = 24.9 %, [95 % CI: 24.7 - 25.1]) was the most important attribute [32]. This was followed by a reduction in the risk of bone pain (RI = 23.2 %, [95 % CI: 23.0 - 23.5]). The authors' concluded that men were more concerned by quality-of-life secondary to side effects than any potential extension in survival [32].

Metastatic Hormone Sensitive Prostate Cancer

A single DCE in mHSPC has been performed by de Freitas and colleagues on a mixed cohort of 152 men, of which a minority (45/152; 29.6%) had mHSPC. The group evaluated Docetaxel or Abiraterone Acetate in combination with ADT. Within the mHSPC sub-group the primary ranked attribute was drug effectiveness on survival (Relative attribute importance (RAI) = 9.08), followed by pain control (RAI = 6.37). Of note, men in this cohort were least concerned about method of treatment delivery (RAI = 1.51) [28].

To our knowledge there have been no attempts in this cohort of men to perform a DCE on systemic therapy in combination with either local cytoreductive and/or metastasis-directed treatments.

STUDY HYPOTHESIS

The null hypothesis is that men with *de novo* synchronous hormone-sensitive metastatic prostate cancer will accept the complications and side-effects associated with combined systemic and cytoreductive local prostate treatment and/or metastasis-directed therapy for any potential oncological benefit.

For peer review only - http://bmjopen.bmj.com/site/about/guidelines.xhtml

PATIENT AND PUBLIC INVOLVEMENT

A patient-involvement interview was held with two patients who had metastatic prostate cancer to determine initial patient acceptability of the study. The integrated qualitative component of this study (**Phase 1 & 2**) incorporates patient-involvement. A patient and public involvement (PPI) representative was present during the HRA REC assessment. This representative will continue to be involved throughout the duration of the study. This will include the Trial Management Group (TMG). Furthermore, other patients that are not involved directly in this study will be present on the independent Trials Steering Committee (TSC).

METHODS AND ANALYSIS

STUDY DESIGN

IP5-MATTER will be performed at sites within the United Kingdom (UK). This will be a cross-sectional questionnaire study. The initial qualitative phase (**Phase 1**) will involve semi-structured interviews with (up to 5) physicians and (up to 5) patients to identify and define the key attributes associated with treatment options that would warrant trade-off evaluation. These will be used to create the first version of the questionnaire (**Phase 1**) [25].

Interviews with participants will be audio-recorded and transcribed verbatim. These will be destroyed on transcription or within two months (whichever occurs earliest). Transcripts will be analysed using an inductive thematic analysis by at least two researchers. Common themes will be extracted by researchers individually and then discussed and agreed upon. The responses and suggestions from the interviews will be used to develop the questionnaire in order to ensure that 1) any attribute or levels that do not seem appropriate are removed or modified; 2) any additional attributes or levels that are not in the questionnaire are included; 3) the questionnaire is understandable; and 4) any complex treatment information is explained in a accessible manner [25].

In the DCE questionnaire, the attributes will be combined into different hypothetical scenarios that will be presented to men in the form of a treatment choice. The hypothetical scenarios included in the questionnaire will be selected using

For peer review only - http://bmjopen.bmj.com/site/about/guidelines.xhtml

experimental design theory. The questionnaire will be trialled on a different group of up to 10 men using a semi-structured 'Think Aloud' interview and any further changes made to create the final version of the DCE questionnaire (**Phase 2**) [33].

This final questionnaire will then be used in the main phase of the study in 300 men across a number of UK trial sites over 12 months (**Phase 3**).

STUDY POPULATION

Men diagnosed with *de novo* synchronous metastatic hormone-sensitive prostate cancer who have not consented to a form of local cytoreductive or metastasis-directed therapy.

ELIGIBILITY

INCLUSION CRITERIA

1. Diagnosed with metastatic hormone-sensitive prostate cancer within 4 months of screening visit.
2. WHO performance status 0-2.

EXCLUSION CRITERIA

1. Castrate-resistant metastatic prostate cancer.
2. Patient has consented to a form of local cytoreductive treatment to the prostate.
3. Patient has consented to a form of metastasis-directed therapy.

IDENTIFICATION OF PATIENTS & CONSENT

All men diagnosed with prostate cancer discussed at the multidisciplinary team (MDT) meeting (or a tumour board) as well as any man meeting the eligibility criteria prior to the MDT meeting will be identified for screening. Members of the MDT will identify patients suitable for IP5-MATTER. The treating clinicians will mention the study and the local Research Nurses, Research Fellows, Clinical Trial Coordinators, Clinical Trial Practitioners or the treating Clinicians will then approach the patient if they are interested.

For peer review only - http://bmjopen.bmj.com/site/about/guidelines.xhtml

For peer review only

Patients with confirmed metastatic prostate cancer and who satisfy the entry criteria will be approached for study enrolment. Patients can be included even if they have already commenced ADT, chemotherapy or an alternative hormonal agent as part of their standard of care. They will be provided with a Patient Information Sheet (PIS) and as much time as they might need to consider whether or not they wish to participate (minimum 24 hours).

Patients already aware of their diagnosis can be approached by telephone to enquire as to their interest in the study so that a PIS can be sent out by email or post prior to a clinical visit.

In the qualitative phases (**Phases 1 & 2**), patients who wish to participate after reading the PIS will be given a date for the semi-structure interview (face-to-face or using audio-visual software). In the main **Phase 3** component, patients who meet the eligibility criteria and are willing to participate will be given the questionnaire to fill in their own time either in the face-to-face or virtual hospital clinic or in their own home. Remote informed consent can be undertaken with the assistance of a telephone or audio-visual software, determined by local availability and protocol. An electronic method (in-built REDCap e-consent form mirroring paper version with 'advanced electronic signature' using a smartphone, tablet, computer) will enable remote informed consent in accordance with HRA-MHRA e-consent guidance (2018) [34]. Informed consent will be signed before implementation of any study-related procedure.

RANDOMISATION

No randomisation is required for this study.

STUDY OUTCOME MEASURES

PRIMARY OUTCOMES

- To determine the attributes associated with treatment that are most important to men with metastatic hormone-sensitive prostate cancer.

For peer review only - http://bmjopen.bmj.com/site/about/guidelines.xhtml

- To determine men's preferences for, and trade-offs between, the attributes (survival and side-effects) of different treatment options in metastatic prostate cancer including systemic therapy, local cytoreductive and metastases-directed therapies.

SECONDARY OUTCOMES

- To inform treatment pathways that are being developed nationally and internationally following randomised trials that are being conducted to evaluate these questions.

- The effect size from these studies can be used with our IP5-MATTER study to determine if, on average, men are willing to accept the potential effect sizes that are demonstrated in trials.

STUDY VISITS

- **Phase 1 or 2**: Single-visit for semi-structured interview or review of electronic study-developed questionnaire (**Table 1**).

- **Phase 3** (Main Study): Single-visit Completion of the electronic DCE during a routine hospital visit or at patient's own home (**Table 1**).

FOLLOW-UP

There is no planned follow-up for this study.

SAMPLE SIZE CALCULATION AND STATISTICAL ANALYSES

Sample Size and Power Considerations

IP5-MATTER trial sample size is set at 300. This is based on the premise that the precision of DCE parameter estimates improves until a sample size of 300 and then

For peer review only - http://bmjopen.bmj.com/site/about/guidelines.xhtml

further gains in precision are small, according to the sampling strategy recommendations set by the International Society for Pharmacoeconomics and Outcomes Research (ISPOR) Good Research Practices for Conjoint Analysis Task Force (2011) [23].

Planned Recruitment Rate

We are aware from the IP2-ATLANTA trial that Centres have approximately 3 men diagnosed with metastatic prostate cancer per month [12, 35]. The initial qualitative phase will focus on the Northwest London regional cancer network (three hospitals) and take 6 weeks to complete. Full phase recruitment is based on 300 patients across 30 sites recruiting a minimum of 10 patients per year or about 1 patient per month. This is an estimated recruitment rate of 26%. Our previous DCEs (i.e. COMPARE) had an acceptance rate of over 50% [31].

Statistical Analysis

Responses from the DCE will be analysed using multinomial logit (MNL) regression techniques that allow for multiple observations from each respondent and preference differences across respondents [25, 31]. This will be used to understand treatment preferences and trade-offs made by patients when considering treatment options of mHSPC. In the MNL analysis, the functional form is specified as:

$$U_{ntj} = \beta X_{ntj} + \varepsilon_{ntj}$$

which represents the utility of option *j* in choice task *t* for patient *n,* where X_{ntj} is a vector of variables representing the levels of the treatment presented in option *j,* β is a vector of utility weights associated with each attribute and ε_{ntj} is the error term. In the analysis, we will assume that patients gain utility (welfare) from treatment and the amount of welfare depends on the attributes and levels of the treatment. We assume that in each choice set patients choose the treatment that would bring them the highest welfare.

For peer review only - http://bmjopen.bmj.com/site/about/guidelines.xhtml

From the results of the DCE, it is possible to estimate the relative importance of attributes and the trade-offs that individuals are willing to make between attributes of a treatment. These trade-offs are how much of one attribute respondents are willing to sacrifice for improvements in another [25].

In this case, we can explore how much life expectancy patients must gain before they are willing to accept treatment side effects. Further, based on the regression results, we can calculate whether the attribute had a positive or negative effect on respondent utility, and the relative importance of a unit change in each attribute when respondents are choosing between treatments. We can also calculate the trade-offs that respondents make between treatment attributes when making choices, which is represented by the ratio of the coefficients [31].

Additionally, we will explore observed preference heterogeneity according to patients' characteristics. The observed characteristics will be analysed by interacting them with the attributes in the MNL model specifications.

DATA COLLECTION

The principal means of data collection from participants will be Electronic Data Capture (EDC) using the web-based REDCap generated platform, provided by the Sponsor. All eCRFs will be completed using de-identified data.

DATA MONITORING & ARCHIVING

On two occasion per year a combined independent data monitoring and trial steering committee will meet. All trial documentation will be archived for a minimum of 10 years following the completion of the study.

TRIAL FUNDING, ORGANISATION AND ADMINISTRATION

IP5-MATTER was approved by Health Research Authority (HRA) East of England, Cambridgeshire and Hertfordshire Research Ethics Committee (20/EE/0194). The study is a joint collaboration between Imperial College London and Health Economic Research Unit (HERU), University of Aberdeen. This study is funded by the Wellcome

For peer review only - http://bmjopen.bmj.com/site/about/guidelines.xhtml

Trust (204998/Z/16/Z) & University College London Hospitals (UCLH) Charity (P83624/1348).

The study will be monitored periodically by trial monitors to assess progress and verify adherence to the protocol, ICH GCP E6 guidelines and other national/international requirements and to review the completeness, accuracy and consistency of the data (**Supplementary Material**). The study may be subject to inspection and audit by regulatory bodies to ensure adherence to GCP and the UK Policy Framework for Health and Social Care Research.

DISCUSSION

IP5-MATTER is a multicentre, prospective discrete choice experiment in men with a new diagnosis of *de novo* synchronous mHSPC. This is the first discrete choice experiment, to the author's knowledge, that evaluates men's treatment preferences for local cytoreductive and metastasis-directed therapy following such a diagnosis.

This study will determine men's preferences for, and trade-offs between, the attributes (survival and side-effects) of different treatment options in metastatic prostate cancer including systemic therapy, local cytoreductive and metastases-directed therapies.

When taken collectively, it is expected that the results will be used to determine if, on average, men are willing to accept the potential effect sizes that are reported in current and future randomised studies. This will have wide-reaching implications on the investment in, and provision of, specific treatment options proposed for integration into the current standard of care treatment pathways for men with mHSPC.

CONCLUSION

IP5-MATTER answers important questions relating to men's decision-making in relation to local cytoreductive and metastasis-directed treatments following a new diagnosis of metastatic hormone-sensitive prostate cancer.

For peer review only

For peer review only - http://bmjopen.bmj.com/site/about/guidelines.xhtml

TRIAL STATUS

IP5-MATTER is planning to open to recruitment in 30 centres in England, Wales and Scotland in January 2021 and planned recruitment completed by January 2022 and study completion in April 2022.

Acknowledgements: We would like to thank all the participants, study PIs, trial clinicians, research nurses, Imperial Clinical Trial Unit staff and other site staff who have been responsible for setting up, recruiting participants and collecting the data for the IP5-MATTER trial. We are also grateful for the ongoing support of the Trial Management Group and our IP5-MATTER patient representatives. Finally, we would like to thank our trial funder the Wellcome Trust and University College Hospitals London (UCLH) Charity.

Contributors: Conception and design of IP5-MATTER trial: MJC, MG, FHSJ, MW, VW, HUA. All authors have read and approved the final manuscript. MJC, MGG, MG, NS, KTJ, GH, FHJ, NKN, JS, TP, DB, AR, MB, BR, SM, VK, TD, AF, MW, VW, HUA.

Patient consent for publication: Not required.

Conflicts of Interest

Martin J. Connor's research is support by University College London Hospitals (UCLH) Charity and the Wellcome Trust.

Mesfin Genie and Verity Watson are based at the Health Economics Research Unit (HERU), University of Aberdeen. HERU is funded by the Chief Scientists Office of the Scottish Government Health and Social Care Directorate.

Kamalram Thippu Jayaprakash acknowledges research grant from the UK National Institute of Health Research Clinical Research Network Eastern and has received educational grants from Bayer UK, Janssen Oncology, Pfizer, Roche, and Takeda.

Hashim U. Ahmed's research is supported by core funding from the United Kingdom's National Institute of Health Research (NIHR) Imperial Biomedical Research Centre. Ahmed currently receives funding from the Wellcome Trust, Prostate Cancer UK, MRC

For peer review only

(UK), Cancer Research UK, The Urology Foundation, BMA Foundation, Imperial Healthcare Charity, Sonacare Inc., Trod Medical, and Sophiris Biocorp for trials in prostate cancer. Ahmed was a paid medical consultant for Sophiris Biocorp, Sonacare Inc. and BTG in the past 3 years. He is currently a paid proctor for Sonacare and Boston.

All other authors have no declaration

References

1 Connor MJ, Shah TT, Horan G, et al. Cytoreductive treatment strategies for de novo metastatic prostate cancer, *Nature Reviews Clinical Oncology* 2019:1-15.

2 Miyake H, Matsushita Y, Watanabe H, et al. Prognostic significance of time to castration resistance in patients with metastatic castration-sensitive prostate cancer, *Anticancer Res* 2019;39:1391-6.

3 Marchioni M, Di Nicola M, Primiceri G, et al. New antiandrogen compounds compared to docetaxel for metastatic hormone sensitive prostate cancer: results from a network meta-analysis, *J Urol* 2020;203:751-9.

4 Sweeney CJ, Chen Y, Carducci M, et al. Chemohormonal therapy in metastatic hormone-sensitive prostate cancer, *N Engl J Med* 2015;373:737-46.

5 Fizazi K, Tran N, Fein L, et al. Abiraterone plus prednisone in metastatic, castration-sensitive prostate cancer, *N Engl J Med* 2017;377:352-60.

6 James ND, de Bono JS, Spears MR, et al. Abiraterone for prostate cancer not previously treated with hormone therapy, *N Engl J Med* 2017;377:338-51.

7 Chi KN, Agarwal N, Bjartell A, et al. Apalutamide for metastatic, castration-sensitive prostate cancer, *N Engl J Med* 2019;381:13-24.

8 James ND, Sydes MR, Clarke NW, et al. Addition of docetaxel, zoledronic acid, or both to first-line long-term hormone therapy in prostate cancer (STAMPEDE): survival results from an adaptive, multiarm, multistage, platform randomised controlled trial, *Lancet* 2016;387:1163-77 doi:10.1016/S0140-6736(15)01037-5 [doi] [published Online First: March 19].

9 James ND, Spears MR, Clarke NW, et al. Survival with newly diagnosed metastatic prostate cancer in the “docetaxel era”: data from 917 patients in the control arm of the STAMPEDE trial (MRC PR08, CRUK/06/019), *Eur Urol* 2015;67:1028-38.

10 Connor MJ, Smith A, Miah S, et al. Targeting Oligometastasis with Stereotactic Ablative Radiation Therapy or Surgery in Metastatic Hormone-sensitive Prostate

For peer review only

For peer review only - http://bmjopen.bmj.com/site/about/guidelines.xhtml

Cancer: A Systematic Review of Prospective Clinical Trials, *European Urology Oncology* 2020.

11 De Bruycker A, Tran PT, Achtman AH, et al. Clinical perspectives from ongoing trials in oligometastatic or oligorecurrent prostate cancer: an analysis of clinical trials registries, *World J Urol* 2020:1-10.

12 ClinicalTrials.gov [Internet]. Additional Treatments to the Local Tumour for Metastatic Prostate Cancer: Assessment of Novel Treatment Algorithms (IP2-ATLANTA). NCT03763253. . 2018;2020.

13 ClinicalTrials.gov [Internet]. Androgen Deprivation Therapy or Androgen Deprivation Therapy Plus Definitive Treatment (Radiation or Surgery) FUSCC-OMPCa. NCT02742675. 2016;2020.

14 ClinicalTrials.gov [Internet]. Best Systemic Therapy or Best Systemic Therapy (BST) Plus Definitive Treatment (Radiation or Surgery). NCT01751438. 2012;2020.

15 ClinicalTrials.gov [Internet]. Standard Systemic Therapy With or Without Definitive Treatment in Treating Participants With Metastatic Prostate Cancer (SWOG 1802). NCT03678025. . 2018;2020.

16 ClinicalTrials.gov [Internet]. Impact of Radical Prostatectomy as Primary Treatment in Patients With Prostate Cancer With Limited Bone Metastases (g-RAMPP). NCT02454543. 2015;2020.

17 ClinicalTrials.gov [Internet]. Cytoreductive Prostatectomy Versus Cytoreductive Prostate Irradiation as a Local Treatment Option for Metastatic Prostate Cancer: a Multicentric Feasibility Trial (LoMP II). NCT03655886. 2018;2020.

18 ClinicalTrials.gov [Internet]. Testing Radical Prostatectomy in Chinese Men With Prostate Cancer and oligoMetastases to the Bone. NCT03988686. 2019;2020.

19 Weichselbaum RR, Hellman S. Oligometastases revisited, *Nature reviews Clinical oncology* 2011;8:378.

20 Tosoian JJ, Gorin MA, Ross AE, et al. Oligometastatic prostate cancer: definitions, clinical outcomes, and treatment considerations, *Nature Reviews Urology* 2017;14:15.

21 Khoo V. New concepts in prostate cancer management: the conundrum of managing oligometastatic disease in prostate cancer—through the looking glass darkly, *Clin Radiol* 2019.

22 Parker CC, James ND, Brawley CD, et al. Radiotherapy to the primary tumour for newly diagnosed, metastatic prostate cancer (STAMPEDE): a randomised controlled phase 3 trial, *Lancet* 2018;392:2353-66 doi:S0140-6736(18)32486-3

23 Bridges JF, Hauber AB, Marshall D, et al. Conjoint analysis applications in health—a checklist: a report of the ISPOR Good Research Practices for Conjoint Analysis Task Force, *Value in health* 2011;14:403-13.

For peer review only

For peer review only - http://bmjopen.bmj.com/site/about/guidelines.xhtml

24 Johnson FR, Lancsar E, Marshall D, et al. Constructing experimental designs for discrete-choice experiments: report of the ISPOR conjoint analysis experimental design good research practices task force, *Value in health* 2013;16:3-13.

25 Lancsar E, Louviere J. Conducting discrete choice experiments to inform healthcare decision making, *Pharmacoeconomics* 2008;26:661-77.

26 de Bekker-Grob EW, Bliemer M, Donkers B, et al. Patients' and urologists' preferences for prostate cancer treatment: a discrete choice experiment, *Br J Cancer* 2013;109:633-40.

27 King MT, Viney R, Smith DP, et al. Survival gains needed to offset persistent adverse treatment effects in localised prostate cancer, *Br J Cancer* 2012;106:638-45.

28 de Freitas HM, Ito T, Hadi M, et al. Patient Preferences for Metastatic Hormone-Sensitive Prostate Cancer Treatments: A Discrete Choice Experiment Among Men in Three European Countries, *Adv Ther* 2019;36:318-32.

29 Eliasson L, de Freitas HM, Dearden L, et al. Patients' preferences for the treatment of metastatic castrate-resistant prostate cancer: A discrete choice experiment, *Clin Ther* 2017;39:723-37.

30 Sculpher M, Bryan S, Fry P, et al. Patients' preferences for the management of non-metastatic prostate cancer: discrete choice experiment, *BMJ* 2004;328:382.

31 Watson V, McCartan N, Krucien N, et al. Evaluating the Trade-offs Men with Localized Prostate Cancer Make between the Risks and Benefits of Treatments: The COMPARE Study, *J Urol* 2020:10.1097/JU. 0000000000000754.

32 Uemura H, Matsubara N, Kimura G, et al. Patient preferences for treatment of castration-resistant prostate cancer in Japan: a discrete-choice experiment, *BMC urology* 2016;16:63.

33 Eccles DW, Arsal G. The think aloud method: what is it and how do I use it? *Qualitative Research in Sport, Exercise and Health* 2017;9:514-31.

34 Health Research Authority. Joint statement on seeking consent by electronic methods. *Health Research Authority (HRA)* 2018.

35 Connor MJ, Shah TT, Sukumar J, et al. Initial experience of the adjuvant treatments to the local tumor for metastatic prostate cancer: Assessment of novel treatment algorithms, a multicenter, phase II randomized controlled trial (IP2-ATLANTA). *Journal of Clinical Oncology* 2020.

For peer review only - http://bmjopen.bmj.com/site/about/guidelines.xhtml

For peer review only

Table and Figure Legends

Table 1. Visit Schedule. *Legend: This study does not have a follow-up visit.*

Figure 1. Example choice task. *Legend: Example choice task for this study that is subject to change following the completion of Phase I and Phase II.*

For peer review only

For peer review only - http://bmjopen.bmj.com/site/about/guidelines.xhtml

Table 1. Visit Schedule

**Visit**	**Screening**	**Visit 1**
**Week**	**0**	**0**
**Informed Consent**	X	
**Inclusion & Exclusion**	X	
**Demographics**	X	
**Medical History**	X	
**Study Intervention: Discrete Choice Experiment**		X

Legend: This study does not have a follow-up visit.

For peer review only - http://bmjopen.bmj.com/site/about/guidelines.xhtml

TREATMENT FEATURES	Treatment A	Treatment B
How your metastatic prostate cancer is managed	Hormonal therapies (with or without chemotherapy)<br><br>followed by **no additional treatment** to prostate	Hormonal therapies (with or without chemotherapy)<br><br>followed by **an overnight-stay surgery** and **4 weeks** of recovery time
Is specialised radiotherapy to cancer deposits included?	No	Yes, there are hospital appointments every day for one week to receive this specialist radiotherapy.
How long a man, on average, is expected to live after the diagnosis?	50 months	70 months
How long, on average, until the cancer starts to grow again?	40 months	60 months
Proportion of men who have permanent urinary incontinence after treatment (%)	5% (5 out of 100)<br>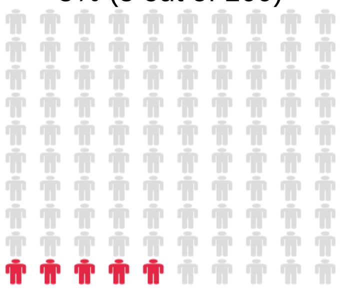	20% (20 out of 100)<br>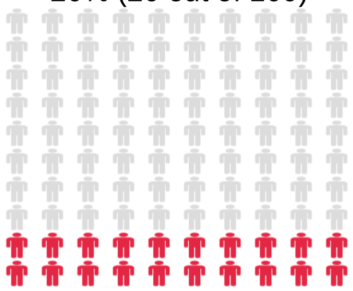
The proportion of men who are <u>not</u> able to maintain an erection sufficient for intercourse (%)	40% (40 out of 100)<br>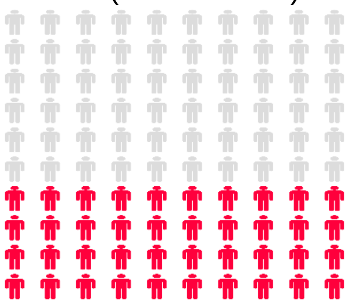	70% (70 out of 100)<br>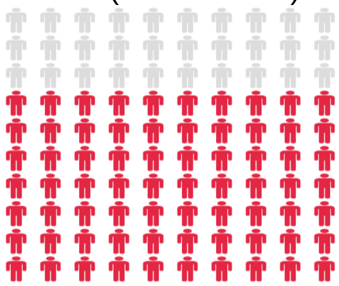
The proportion of men who have a feeling of extreme tiredness (fatigue) impacting on daily activities (%)	10% (10 out of 100)<br>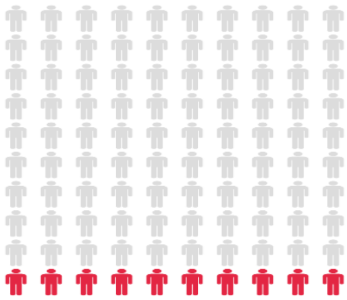	40% (40 out of 100)<br>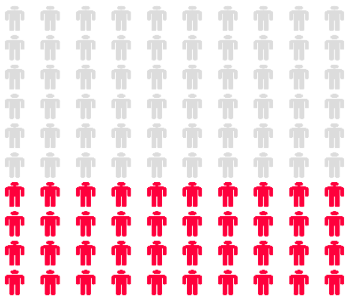
**Which treatment option would you choose?**	☐	☐

SUPPLEMENTARY MATERIAL

1. FULL STUDY DETAILS

Full Study Title: Patients' preferences in the treatment of hormone-sensitive metastatic prostate cancer: a discrete choice experiment

Short Study title / Acronym: Metastatic prostate cancer men's Attitudes towards Treatment of the local Tumour and metastasis Evaluative Research (**IP5-MATTER**)

Product: Discrete Choice Experiment

Development Phase: Multicentre Observational Study

Sponsor: Imperial College London

Version no: 2.0

Protocol Date: 27th August 2020

**IRAS ID:**	276834
**REC Reference Number:**	20/EE/0194
**Clinical trials.gov Number:**	NCT04590976
**Sponsor Protocol Number:**	20CX5760
**Funder reference:**	Wellcome Trust. Senior Clinical Fellowship. Ref: 204998/Z/16/Z. University College Hospitals London (UCLH) Charity (P83624/1348)

2. REGULATORY, ETHICAL AND LEGAL ISSUES

2.1 Declaration of Helsinki

The investigator will ensure that this study is conducted in full conformity with the 7th revision of the 1964 Declaration of Helsinki.

2.2 Good Clinical Practice

The study will be conducted in accordance with the guidelines laid down by the International Conference on Harmonisation for Good Clinical Practice (ICH GCP E6 guidelines).

2.3 Independent Ethics Committee Approval

(i) Initial Approval

Prior to the enrolment of subjects, the REC must provide written approval of the conduct of the study at named sites, the protocol and any amendments, the Patient Information Sheet and Consent Form, any other written information that will be provided to the subjects, any advertisements that will be used and details of any subject compensation.

For peer review only

(ii) Approval of Amendments

Proposed amendments to the protocol and aforementioned documents must be submitted to the REC for approval as instructed by the Sponsor. Amendments requiring REC approval may be implemented only after a copy of the REC's approval letter has been obtained.

Amendments that are intended to eliminate an apparent immediate hazard to subjects may be implemented prior to receiving Sponsor or REC approval. However, in this case, approval must be obtained as soon as possible after implementation.

(iii) Annual Progress Reports

The REC will be sent annual progress reports in accordance with national requirements.

(iv) Annual Progress Reports and End of Trial Notification

The REC will be sent annual progress updates in order to facilitate their continuing review of the study (reference. ICH GCP E6 Section 3.1.4) and will also be informed about the end of the trial, within the required timelines. The Annual Progress Report will detail all SAEs recorded.

2.4 HRA approval

Health Research Authority (HRA) approval will be obtained prior to starting the study. Each participating site will confirm capacity and capability prior to commencing.
The HRA and all participating sites also need to be notified of all protocol amendments to assess whether the amendment affects the institutional approval for each site.

2.5 Other Required Approvals

None

2.6 Non-Compliance and Serious Breaches

All protocol deviations and protocol violations will be reported via MATTER trial team email address and reviewed by the Principal Investigator and reported to the trial TMG on a monthly basis. Protocol violations will be reported to the Sponsor.

An assessment of whether the protocol deviation/violation constitutes a serious breach will be made.

A serious breach is defined as:

A breach of the conditions and principles of GCP in connection with a trial or the trial protocol, which is likely to affect to a significant degree:

- The safety or physical or mental integrity of the UK trial subjects; or
- The overall scientific value of the trial

For peer review only

The Sponsor will be notified within 24 hours of identifying a likely Serious Breach. If a decision is made that the incident constitutes a Serious Breach, this will be reported to the REC within 7 days of becoming aware of the serious breach.

2.7 Insurance and Indemnity

Imperial College London holds negligent harm and non-negligent harm insurance policies, which apply to this study. *Imperial College London will act as the main Sponsor for this study. Delegated responsibilities will be assigned to the participating sites taking part in this study.*

2.8 Trial Registration

The study will be registered on a trial database e.g. ClinicalTrials.gov in accordance with requirements of the International Committee of Medical Journal Editors (ICMJE) regulations.

2.9 Informed Consent

Subjects will be provided with a copy of the signed Subject Information Sheet/Informed Consent Form document. The original Informed Consent Form should be retained with the source documents.

2.10 Contact with General Practitioner

It is the investigator's responsibility to inform the subject's General Practitioner (where applicable) by letter that the subject is taking part in the study provided the subject agrees to this, and information to this effect is included in the Subject Information Sheet and Informed Consent. A copy of the letter should be filed in the medical notes.

2.11 Subject Confidentiality

The investigator must ensure that the subject's confidentiality is maintained. On the CRF or other documents submitted to the Sponsors, subjects will be identified by a subject ID number only. Documents that are not submitted to the Sponsor (e.g., signed informed consent form) should be kept in a strictly confidential file by the investigator.

The investigator shall permit direct access to subjects' records and source documents for the purposes of monitoring, auditing, or inspection by the Sponsor, authorised representatives of the Sponsor and RECs.

2.12 Data Protection and Patient Confidentiality

The investigator will preserve the confidentiality of all participants taking part in the study, which will be conducted in accordance with GDPR and the Data Protection Act. Furthermore, all investigators and study staff must comply with the requirements of the Data Protection Act 2018 with regards to the collection, storage, processing and disclosure of personal information and will uphold the Act's core principles.

2.13 End of Trial

The end of the trial will be marked by the completion of the study report so that all study outcomes can be addressed. However, the trial may end early upon recommendation of the Trial Steering Committee.

For peer review only

2.14 Study Documentation and Data Storage

The study investigators and study site staff will comply with the requirements of the Data Protection Act 2018 concerning the collection, storage, processing and disclosure of personal information and will uphold the Act's core principles.

The investigator must retain essential documents until notified by the Sponsor, and for at least ten years after study completion. Subject files and other source data (including copies of protocols, CRFs, original reports of test results, correspondence, records of informed consent, and other documents pertaining to the conduct of the study) must be retained. Documents should be stored in such a way that they can be accessed/data retrieved at a later date.

All research documentation and information will undergo a process of pseudonymisation where possible. Whilst within the study, patients will be identified by a unique study number, and the data in the CRF will be linked to this number. Research data will be entered onto a dedicated, secure, encrypted trial database, specifically constructed for this purpose. The study team will maintain the confidentiality of all patient data and will not disclose information by which patients may be identified to any third party other than those directly involved in the treatment of the patient and organisations for which the patient has given explicit consent for data transfer. Data within the NHS system such as patient notes, and reports will remain confidential in accordance with NHS confidentiality code of practice.

Paper enrolment logs, including patients' names, NHS numbers and dates of birth, will be kept in the Investigator Site File and will be stored in a secure, locked room within each participating site. Electronic documents will be kept on secure and encrypted computers. Access to these documents will be highly restricted and will only be available to the relevant research team members.

No study documents will be destroyed without prior written agreement between the Sponsor and the investigator. Should the investigator wish to assign the study records to another party or move them to another location, written agreement must be obtained from the Sponsor.

However, psuedonymised data will be shared with Dr Verity Watson at the Health Economics Research Unit (HERU), University of Aberdeen, Aberdeen for analysis at completion of the study.

3. STUDY WITHDRAWAL

3.1 Permanent Discontinuation of Study Treatment and Withdrawal from Study

(v) Permanent discontinuation of study treatment

Subjects may discontinue study treatment for the following reasons:

- At the request of the subject.
- Adverse event/ Serious Adverse Event
- If the investigator considers that a subject's health will be compromised due to adverse events or concomitant illness that develop after entering the study.

(vi) Withdrawal from Study

Withdrawal from the study refers to discontinuation of study treatment and study procedures and can occur for the following reasons:

- Subject decision

For peer review only

- Loss to follow-up

3.2 Adverse Events

DEFINITIONS

Adverse Event (AE): any untoward medical occurrence in a patient or clinical study subject.

Serious Adverse Event (SAE): any untoward and unexpected medical occurrence or effect that:

- **Results in death**
- **Is life-threatening** – *refers to an event in which the subject was at risk of death at the time of the event; it does not refer to an event which hypothetically might have caused death if it were more severe*
- **Requires hospitalisation, or prolongation of existing inpatients' hospitalisation**
- **Results in persistent or significant disability or incapacity**
- **Is a congenital anomaly or birth defect**

Medical judgement should be exercised in deciding whether an AE is serious in other situations. Important AEs that are not immediately life-threatening or do not result in death or hospitalisation but may jeopardise the subject or may require intervention to prevent one of the other outcomes listed in the definition above, should also be considered serious.

REPORTING PROCEDURES

All adverse events should be reported. Depending on the nature of the event the reporting procedures below should be followed. Any questions concerning adverse event reporting should be directed to the Chief Investigator in the first instance.

Non serious AEs
All such events, whether expected or not, should be recorded.

Serious AEs
An SAE form should be completed on RedCap and also an email sent to the Chief Investigator within 24 hours. However, relapse and death due to metastatic prostate cancer, and hospitalisations for elective treatment of a pre-existing condition do not need reporting as SAEs.

All SAEs should be reported to the **HRA East of England, Cambridgeshire and Hertfordshire Research Ethics Committee (20/EE/0194)** where in the opinion of the Chief Investigator, the event was:

- 'related', ie resulted from the administration of any of the research procedures; and
- 'unexpected', ie an event that is not listed in the protocol as an expected occurrence

Reports of related and unexpected SAEs should be submitted within 15 days of the Chief Investigator becoming aware of the event, using the NRES SAE form for non-IMP studies. The Chief Investigator must also notify the Sponsor of all SAEs.

Local investigators should report any SAEs as required by their Local Research Ethics Committee, Sponsor and/or Research & Development Office

For peer review only

4. DATA MANAGEMENT

4.1 Source Data

Source documentation is defined as the first-time data appears, and may include original document, data and records (hospital records, clinical reports, MRI and Ultrasound reports, other procedure reports, laboratory notes, other data recorded at the pathology and biochemistry laboratories, etc.). Information in source documents (e.g. medical history) dated prior to the Informed Consent Form signature date may be used to verify patient suitability for the study.

Clinical records must be marked to indicate a subject has been enrolled into the clinical study.

The Investigator must ensure the availability of source documents from which the information on the eCRF was obtained. Where printouts and electronic medical records are provided as source documents, they should be signed and dated by a member of the adequately trained research team, to indicate that the data provided is a true reproduction of the original source document.

All study data may be inspected by the Sponsor and regulatory authorities by people working on behalf of the Sponsor, and by representatives of Regulatory Authorities, where it is relevant to this research.

4.2 Language

CRFs will be in English. Generic names for concomitant medications should be recorded in the CRF wherever possible. All written material to be used by subjects must use vocabulary that is clearly understood and be in the language appropriate for the study site.

4.3 Database

Data collection will be via Electronic Data Capture (EDC) using the online REDCap database. Data is entered into the EDC system via site personnel. All source data will be recorded in the CRF and the completed case report book will be signed by the Investigator or his/her appropriate designee. All changes made at any time following the electronic signing will have an electronic audit trail with a signature and date. The completed case report book will then again be signed by the investigator or his/her appropriate designee. Specific instructions and further details will be outlined in the CRF manual.

4.4 Data Collection

All study data will be entered into electronic Case Report (eCRFs) in a database provided by using REDCap. All eCRFs will be completed using de-identified data.

CRF completion may be delegated by the Principal Investigator (documented on the Delegated Task List) to other study personnel though the Principal Investigator remains responsible for the accuracy and integrity of all data entered to eCRFs. Further details of procedures for CRF/eCRF completion, including data review, database cleaning, issuing and resolving data queries, and identification of steps for creation, modification, maintenance and

For peer review only

archiving of source data via any computerised systems will be provided in the study specific Data Management Plan (CRF manual).

4.5 Archiving

All trial documentation, including that held at participating sites and the trial coordinating centre, will be archived for a minimum of 10 years following the end of the study. Subject files and other source data (including copies of protocols, CRFs, original reports of test results, correspondence, records of informed consent, and other documents pertaining to the conduct of the study) must be retained. Documents should be stored in such a way that they can be accessed/data retrieved later. Consideration should be given to security and environmental risks.

No study document will be destroyed without prior written agreement between the Sponsor and the investigator. Should the investigator wish to assign the study records to another party or move them to another location, written agreement must be obtained from the Sponsor.

Storage and handling of confidential trial data and documents will be in accordance with the Data Protection Act 2018 (UK).

5. STUDY MANAGEMENT STRUCTURE

5.1 Trial Steering Committee

A Trial Steering Committee (TSC) will be convened including as a minimum an Independent Chair, Independent Clinician, the Chief Investigator and Study Manager. The role of the TSC is to provide overall supervision of trial conduct and progress. Details of membership, responsibilities and frequency of meetings will be defined in a separate Charter.

5.2 Trial Management Group

A Trial Management Group (TMG) will be convened including the Principal Investigators, co-investigators and key collaborators, Study Statistician and Study Manager. The TMG will be responsible for day-to-day conduct of the trial and operational issues. Details of membership, responsibilities and frequency of meetings will be defined in separate Terms of Reference. Authorship of publications will be determined by the TMG. When necessary, decisions will be referred to the TSC. Meetings will be scheduled in a risk-adapted manner to allow for the review of events during the trial.

5.3 Data Monitoring Committee (DMC)

A combined data monitoring and trial steering committee will meet twice a year basis. The composition of this committee will include but not be limited to the Chief Investigator, Trial Statistician, Trial Coordinator, Research nurse and Patient representative.

5.4 Early Discontinuation of the Study

The following reasons may result in early discontinuation:

- It is not feasible to reach the planned outcomes (e.g. recruitment rate will not reach 300).

The statistical criteria for termination of the study will be detailed in the statistical analysis plan (SAP).

5.5 Risk Assessment

A study-specific risk assessment will be performed prior to the start of the study to assign a risk category of 'low', 'medium' or 'high' to the trial. Risk assessment will be carried out by Prof Hashim Ahmed and Mr Martin Connor in collaboration with the TMG the result will be used to guide a monitoring plan. The risk assessment will consider all aspects of the study and will be updated as required during the course of the study.

5.6 Monitoring

The study will be monitored periodically by trial monitors to assess the progress of the study, verify adherence to the protocol, ICH GCP E6 guidelines and other national/international requirements and to review the completeness, accuracy and consistency of the data.

Monitoring procedures and requirements will be documented in a Monitoring Plan, developed in accordance with the risk assessment.

5.7 Quality Control and Quality Assurance

Quality Control will be performed according to internal procedures. The study may be audited by a Quality Assurance representative of the Sponsor. All necessary data and documents will be made available for inspection.
The study may be subject to inspection and audit by regulatory bodies to ensure adherence to GCP and the UK Policy Framework for Health and Social Care Research.

5.8 Peer review

This study has been peer reviewed by The Urology Foundation (TUF), the Royal College of Surgeons of England and the British Medical Association (Appendix A). The study is funded by UCLH Charity, 5th Floor East, 250 Euston Road London, NW1 2PG (Appendix B). It has been reviewed within the Urology Department at Imperial College London and the Health Economics Research Unit, Polwarth Building, University of Aberdeen.

5.9 Patient and Public Involvement

5.10 Publication and Dissemination policy

Information concerning the study, patent applications, processes, scientific data or other pertinent information is confidential and remains the property of the Sponsor. The investigator may use this information for the purposes of the study only.

It is understood by the investigator that the Sponsor will use information developed in this clinical study in connection with the development of the ablative or radiotherapy or surgical techniques and, therefore, may disclose it as required to other clinical investigators and to Regulatory Authorities. In order to allow the use of the information derived from this clinical study, the investigator understands that he/she has an obligation to provide complete test results and all data developed during this study to the Sponsor.

Verbal or written discussion of results prior to study completion and full reporting should only be undertaken with written consent from the Sponsor.

Therefore, all information obtained as a result of the study will be regarded as CONFIDENTIAL, at least until appropriate analysis and review by the investigator(s) are completed.

For peer review only

Permission from the Executive/Writing Committee is necessary prior to disclosing any information relative to this study outside of the Trial Steering Committee. Any request by site investigators or other collaborators to access the study dataset must be formally reviewed by the TMG.

A Clinical Study Report summarising the study results will be prepared and submitted to the REC within a year of the end of study.

For peer review only

6. INFORMED CONSENT FORM

INFORMED CONSENT FORM (Phase 3/Main Study)

**IP5-MATTER**
**M**etastatic prostate cancer men's **A**ttitudes towards **T**reatment of the local **T**umour and metastasis **E**valuative **R**esearch (**IP5-MATTER**)

Chief Investigator: Professor Hashim U. Ahmed

Please initial each box below. Do not tick

1	I confirm that I have read and understand the patient information sheet dated _ _ / _ _ / _ _ _ _ (Version ___) for the **MATTER** Phase 3 / Main Study. I have had the opportunity to consider the information, ask questions and have had these answered satisfactorily.	
2	I understand that my participation is voluntary and that I am free to withdraw at any time, without giving reason and without my medical care or legal rights being affected.	
3	I understand that relevant sections of my medical notes and data collected during the study, may be looked at by individuals from the Sponsor of the trial (Imperial College London) and responsible persons authorised by the Sponsor, from regulatory authorities or from the NHS Trust, where it is relevant to my taking part in this research. I give permission for these individuals to have access to my records.	
4	I understand that I can stop the questionnaire at any time, for any reason.	
5	I understand that the information collected about me will be used to support other ethically approved research in future, and may be shared anonymously with other researchers.	
6	I give permission for the researchers to contact me regarding this trial during this trial period.	
7	I agree for my GP and other doctors to be informed of my participation in this study and of any clinical relevant study results.	
8	I agree to take part in the above study.	

All the boxes above must be initialled for consent to be valid

Site Number: INSERT
Protocol Number: 20CX5760

Version 2.0 27 AUG 2020
IRAS Number: 276834

For peer review only

OPTIONAL CONSENT

Please initial each box below. Do not tick

10	I give consent for information collected about me to be used to support other ethically approved research in the future, including those outside of the EEA	
11	I give consent to being contacted to potentially taking part in other ethically approved research studies.	

Name of Participant Signature Date

Name of Person taking consent Signature Date

Please give one copy of the consent form to the patient, file one copy in the patient's medical records, and retain the original in the Investigator Site File

Site Number: INSERT
Protocol Number: 20CX5760

Version 2.0 27 AUG 2020
IRAS Number: 276834

SPIRIT

STANDARD PROTOCOL ITEMS: RECOMMENDATIONS FOR INTERVENTIONAL TRIALS

SPIRIT 2013 Checklist: Recommended items to address in a clinical trial protocol and related documents*

Page No.	Section/item	Item No	Description
	**Administrative information**		
**1**	Title	1	Descriptive title identifying the study design, population, interventions, and, if applicable, trial acronym
**2**	Trial registration	2a	Trial identifier and registry name. If not yet registered, name of intended registry
**2,3**		2b	All items from the World Health Organization Trial Registration Data Set
**Suppl.**	Protocol version	3	Date and version identifier
**3,14, 15**	Funding	4	Sources and types of financial, material, and other support
**1,2,16**	Roles and responsibilities	5a	Names, affiliations, and roles of protocol contributors
**14,15**		5b	Name and contact information for the trial sponsor
**14,15, Suppl.**		5c	Role of study sponsor and funders, if any, in study design; collection, management, analysis, and interpretation of data; writing of the report; and the decision to submit the report for publication, including whether they will have ultimate authority over any of these activities
**Suppl.**		5d	Composition, roles, and responsibilities of the coordinating centre, steering committee, endpoint adjudication committee, data management team, and other individuals or groups overseeing the trial, if applicable (see Item 21a for data monitoring committee)

For peer review only

	**Introduction**		
**5-8**	Background and rationale	6a	Description of research question and justification for undertaking the trial, including summary of relevant studies (published and unpublished) examining benefits and harms for each intervention
**5-8**		6b	Explanation for choice of comparators
**8**	Objectives	7	Specific objectives or hypotheses
**9,10**	Trial design	8	Description of trial design including type of trial (eg, parallel group, crossover, factorial, single group), allocation ratio, and framework (eg, superiority, equivalence, noninferiority, exploratory)
	**Methods: Participants, interventions, and outcomes**		
**10-11**	Study setting	9	Description of study settings (eg, community clinic, academic hospital) and list of countries where data will be collected. Reference to where list of study sites can be obtained
**10-11**	Eligibility criteria	10	Inclusion and exclusion criteria for participants. If applicable, eligibility criteria for study centres and individuals who will perform the interventions (eg, surgeons, psychotherapists)
**9**	Interventions	11a	Interventions for each group with sufficient detail to allow replication, including how and when they will be administered
**Suppl.**		11b	Criteria for discontinuing or modifying allocated interventions for a given trial participant (eg, drug dose change in response to harms, participant request, or improving/worsening disease)
**11**		11c	Strategies to improve adherence to intervention protocols, and any procedures for monitoring adherence (eg, drug tablet return, laboratory tests)
**10**		11d	Relevant concomitant care and interventions that are permitted or prohibited during the trial
**11-12**	Outcomes	12	Primary, secondary, and other outcomes, including the specific measurement variable (eg, systolic blood pressure), analysis metric (eg, change from baseline, final value, time to event), method of aggregation (eg, median, proportion), and time point for each outcome. Explanation of the clinical relevance of chosen efficacy and harm outcomes is strongly recommended

For peer review only - http://bmjopen.bmj.com/site/about/guidelines.xhtml

**12, Table 1**	Participant timeline	13	Time schedule of enrolment, interventions (including any run-ins and washouts), assessments, and visits for participants. A schematic diagram is highly recommended (see Figure)
**3, 12-13**	Sample size	14	Estimated number of participants needed to achieve study objectives and how it was determined, including clinical and statistical assumptions supporting any sample size calculations
**10-11**	Recruitment	15	Strategies for achieving adequate participant enrolment to reach target sample size
	**Methods: Assignment of interventions (for controlled trials)**		
	Allocation:		
**N/A**	Sequence generation	16a	Method of generating the allocation sequence (eg, computer-generated random numbers), and list of any factors for stratification. To reduce predictability of a random sequence, details of any planned restriction (eg, blocking) should be provided in a separate document that is unavailable to those who enrol participants or assign interventions
**N/A**	Allocation concealment mechanism	16b	Mechanism of implementing the allocation sequence (eg, central telephone; sequentially numbered, opaque, sealed envelopes), describing any steps to conceal the sequence until interventions are assigned
**N/A**	Implementation	16c	Who will generate the allocation sequence, who will enrol participants, and who will assign participants to interventions
**N/A**	Blinding (masking)	17a	Who will be blinded after assignment to interventions (eg, trial participants, care providers, outcome assessors, data analysts), and how
**N/A**		17b	If blinded, circumstances under which unblinding is permissible, and procedure for revealing a participant's allocated intervention during the trial

	**Methods: Data collection, management, and analysis**		
**18**	Data collection methods	18a	Plans for assessment and collection of outcome, baseline, and other trial data, including any related processes to promote data quality (eg, duplicate measurements, training of assessors) and a description of study instruments (eg, questionnaires, laboratory tests) along with their reliability and validity, if known. Reference to where data collection forms can be found, if not in the protocol
**18**		18b	Plans to promote participant retention and complete follow-up, including list of any outcome data to be collected for participants who discontinue or deviate from intervention protocols
**18, Suppl.**	Data management	19	Plans for data entry, coding, security, and storage, including any related processes to promote data quality (eg, double data entry; range checks for data values). Reference to where details of data management procedures can be found, if not in the protocol
**12-13**	Statistical methods	20a	Statistical methods for analysing primary and secondary outcomes. Reference to where other details of the statistical analysis plan can be found, if not in the protocol
**12-13**		20b	Methods for any additional analyses (eg, subgroup and adjusted analyses)
**12, Suppl.**		20c	Definition of analysis population relating to protocol non-adherence (eg, as randomised analysis), and any statistical methods to handle missing data (eg, multiple imputation)
	**Methods: Monitoring**		
**14, Suppl.**	Data monitoring	21a	Composition of data monitoring committee (DMC); summary of its role and reporting structure; statement of whether it is independent from the sponsor and competing interests; and reference to where further details about its charter can be found, if not in the protocol. Alternatively, an explanation of why a DMC is not needed
**14, Suppl.**		21b	Description of any interim analyses and stopping guidelines, including who will have access to these interim results and make the final decision to terminate the trial

For peer review only - http://bmjopen.bmj.com/site/about/guidelines.xhtml

**Suppl.**	Harms	22	Plans for collecting, assessing, reporting, and managing solicited and spontaneously reported adverse events and other unintended effects of trial interventions or trial conduct
**Suppl.**	Auditing	23	Frequency and procedures for auditing trial conduct, if any, and whether the process will be independent from investigators and the sponsor
	**Ethics and dissemination**		
**14-15**	Research ethics approval	24	Plans for seeking research ethics committee/institutional review board (REC/IRB) approval
**Suppl.**	Protocol amendments	25	Plans for communicating important protocol modifications (eg, changes to eligibility criteria, outcomes, analyses) to relevant parties (eg, investigators, REC/IRBs, trial participants, trial registries, journals, regulators)
**10,11**	Consent or assent	26a	Who will obtain informed consent or assent from potential trial participants or authorised surrogates, and how (see Item 32)
**N/A**		26b	Additional consent provisions for collection and use of participant data and biological specimens in ancillary studies, if applicable
**14, Suppl.**	Confidentiality	27	How personal information about potential and enrolled participants will be collected, shared, and maintained in order to protect confidentiality before, during, and after the trial
**16**	Declaration of interests	28	Financial and other competing interests for principal investigators for the overall trial and each study site
**Suppl.**	Access to data	29	Statement of who will have access to the final trial dataset, and disclosure of contractual agreements that limit such access for investigators
**Suppl.**	Ancillary and post-trial care	30	Provisions, if any, for ancillary and post-trial care, and for compensation to those who suffer harm from trial participation

For peer review only

For peer review only - http://bmjopen.bmj.com/site/about/guidelines.xhtml

**3, Suppl.**	Dissemination policy	31a	Plans for investigators and sponsor to communicate trial results to participants, healthcare professionals, the public, and other relevant groups (eg, via publication, reporting in results databases, or other data sharing arrangements), including any publication restrictions
		31b	Authorship eligibility guidelines and any intended use of professional writers
		31c	Plans, if any, for granting public access to the full protocol, participant-level dataset, and statistical code
	**Appendices**		
**Suppl.**	Informed consent materials	32	Model consent form and other related documentation given to participants and authorised surrogates
**N/A**	Biological specimens	33	Plans for collection, laboratory evaluation, and storage of biological specimens for genetic or molecular analysis in the current trial and for future use in ancillary studies, if applicable

*It is strongly recommended that this checklist be read in conjunction with the SPIRIT 2013 Explanation & Elaboration for important clarification on the items. Amendments to the protocol should be tracked and dated. The SPIRIT checklist is copyrighted by the SPIRIT Group under the Creative Commons "Attribution-NonCommercial-NoDerivs 3.0 Unported" license.

For peer review only

BMJ Open

Metastatic prostate cancer men's Attitudes towards Treatment of the local Tumour and metastasis Evaluative Research (IP5-MATTER): Protocol for a prospective, multicentre discrete choice experiment study

Journal:	*BMJ Open*
Manuscript ID	bmjopen-2021-048996.R1
Article Type:	Protocol
Date Submitted by the Author:	23-Aug-2021
Complete List of Authors:	Connor, Martin; Imperial College London, Department of Surgery and Cancer<br>Genie, Mesfin G; University of Aberdeen, Health Economics Research Unit; Ca' Foscari University of Venice, Economics<br>Gonzalez, Michael; Imperial College Healthcare NHS Trust, Department of Oncology<br>Sarwar, Naveed; Imperial College Healthcare NHS Trust, Department of Oncology<br>Thippu Jayaprakash, Kamalram; Addenbrooke's Hospital, Oncology; Queen Elizabeth Hospital King's Lynn NHS Foundation Trust, Oncology<br>Horan, Gail; Addenbrooke's Hospital, Department of Oncology<br>Hosking-Jervis, Feargus; Imperial College London Division of Surgery, Imperial College London<br>Klimowska-Nassar, Natalia; Imperial College London, Department of Surgery and Cancer; Imperial College London, Imperial College Clinical Trials Unit<br>Sukumar, Johanna; Imperial College London, Department of Surgery and Cancer; Imperial College London, Imperial College Clinical Trials Unit<br>Pokrovska, Tzveta; Imperial College Healthcare NHS Trust, Department of Oncology<br>Basak, Dolan; Imperial College Healthcare NHS Trust, Department of Oncology<br>Robinson , Angus ; Brighton and Sussex University Hospitals NHS Trust, Department of Oncology<br>Beresford, Mark; Royal United Hospitals Bath NHS Foundation Trust, Department of Oncology<br>Rai, Bhavan; Newcastle Upon Tyne Hospitals NHS Foundation Trust, Department of Urology<br>Mangar, Stephen; Imperial College Healthcare NHS Trust, Department of Oncology<br>Khoo, Vincent; Royal Marsden NHS Foundation Trust, Department of Oncology<br>Dudderidge, Tim; University Hospital Southampton NHS Foundation Trust, Department of Urology<br>Falconer, Alison; Imperial College Healthcare NHS Trust, Department of Oncology<br>Winkler, Mathias; Imperial College London, Department of Surgery and Cancer; Imperial College Healthcare NHS Trust, Imperial Urology

	Watson, Verity; University of Aberdeen, Health Economics Research Unit (HERU)<br>Ahmed, Hashim; Imperial College London Division of Surgery, Imperial College London
**Primary Subject Heading**:	Urology
Secondary Subject Heading:	Oncology, Health economics, Patient-centred medicine
Keywords:	Prostate disease < UROLOGY, HEALTH ECONOMICS, Urological tumours < ONCOLOGY, RADIOTHERAPY, SURGERY, Radiation oncology < RADIOLOGY & IMAGING

I, the Submitting Author has the right to grant and does grant on behalf of all authors of the Work (as defined in the below author licence), an exclusive licence and/or a non-exclusive licence for contributions from authors who are: i) UK Crown employees; ii) where BMJ has agreed a CC-BY licence shall apply, and/or iii) in accordance with the terms applicable for US Federal Government officers or employees acting as part of their official duties; on a worldwide, perpetual, irrevocable, royalty-free basis to BMJ Publishing Group Ltd ("BMJ") its licensees and where the relevant Journal is co-owned by BMJ to the co-owners of the Journal, to publish the Work in this journal and any other BMJ products and to exploit all rights, as set out in our licence.

The Submitting Author accepts and understands that any supply made under these terms is made by BMJ to the Submitting Author unless you are acting as an employee on behalf of your employer or a postgraduate student of an affiliated institution which is paying any applicable article publishing charge ("APC") for Open Access articles. Where the Submitting Author wishes to make the Work available on an Open Access basis (and intends to pay the relevant APC), the terms of reuse of such Open Access shall be governed by a Creative Commons licence – details of these licences and which Creative Commons licence will apply to this Work are set out in our licence referred to above.

Other than as permitted in any relevant BMJ Author's Self Archiving Policies, I confirm this Work has not been accepted for publication elsewhere, is not being considered for publication elsewhere and does not duplicate material already published. I confirm all authors consent to publication of this Work and authorise the granting of this licence.

For peer review only

Metastatic prostate cancer men's Attitudes towards Treatment of the local Tumour and metastasis Evaluative Research (**IP5-MATTER**): Protocol for a prospective, multicentre discrete choice experiment study

Martin J. Connor [1,2,3], Mesfin G. Genie [4], Michael Gonzalez [5], Naveed Sarwar [5], Kamalram Thippu Jayaprakash [6], Gail Horan [6], Feargus Hosking-Jervis [1], Natalia Klimowska-Nassar [1,7], Johanna Sukumar [1,7], Tzveta Pokrovska [5], Dolan Basak [5], Angus Robinson [8], Mark Beresford [9], Bhavan Rai [10], Stephen Mangar [5], Vincent Khoo [11], Tim Dudderidge [12], Alison Falconer [5], Mathias Winkler [1,2,3], Verity Watson* [4], Hashim U. Ahmed* [1,2]

1. Imperial Prostate, Division of Surgery, Department of Surgery and Cancer, Faculty of Medicine, Imperial College London, London, UB1 3HW
2. Imperial Urology, Imperial College Healthcare NHS Trust, London, W6 8RF
3. Department of Urology, West Middlesex University Hospital (WMUH), Chelsea & Westminster NHS Foundation Trust, Isleworth, Middlesex, TW7 6AF
4. Health Economic Research Unit (HERU), Faculty of Medicine, University of Aberdeen, Aberdeen
5. Department of Oncology, Imperial College Healthcare NHS Foundation Trust, Charing Cross Hospital, London, W6 8RF
6. Department of Oncology, Addenbrooke's Hospital and The Queen Elizabeth Hospital Kings Lynn NHS Foundation, Norfolk, PE30 4ET
7. Imperial Clinical Trials Unit (ICTU), School of Public Health, Faculty of Medicine, Imperial College London, London, UB1 3HW
8. Department of Oncology, Brighton and Sussex University Hospitals NHS Trust, Brighton BN2 1ES
9. Department of Oncology, The Royal United Hospitals Bath, Bath, BA1 3NG
10. Department of Urology, Northern Centre for Cancer Care, Newcastle upon Tyne Hospitals NHS Foundation Trust, NE7 7DN
11. Department of Clinical Oncology, The Royal Marsden Hospital & Institute of Cancer Research, London, SW3 6JJ

For peer review only

For peer review only - http://bmjopen.bmj.com/site/about/guidelines.xhtml

12. Department of Urology, University Hospital Southampton NHS Foundation Trust, Southampton, SO16 6YD

Corresponding Author:
Mr. Martin J. Connor
Division of Surgery
Department of Surgery & Cancer
Imperial College London
Room 5L.28
Charing Cross Lab Block
m.connor@imperial.ac.uk

**Considered joint senior author

For peer review only

Main text: 3,508 Words

Keywords: *prostate cancer, metastatic, radiotherapy, radical prostatectomy, cytoreductive, SABR, SBRT, stereotactic radiotherapy, high intensity focussed ultrasound, cryotherapy, oligometastatic, choice behaviour, health economics*

Abstract

Introduction: Systemic therapy with androgen deprivation therapy (ADT) and intensification with agents such as Docetaxel, Abiraterone Acetate and Enzalutamide, has resulted in improved overall survival in men with *de novo* synchronous metastatic hormone-sensitive prostate cancer (mHSPC). Novel local cytoreductive treatments and metastasis-directed therapy are now being evaluated. Such interventions may provide added survival benefit or delay the requirement for further systemic agents and associated toxicity but can confer additional harm. Understanding men's preferences for treatment options in this disease state is crucial for patients, clinicians, carers and future healthcare service providers.

Methods: Using a prospective, multi-centre discrete choice experiment (DCE), we aim to determine the attributes associated with treatment that are most important to men with mHSPC. Furthermore, we plan to determine men's preferences for, and trade-offs between, the attributes (survival and side-effects) of different treatment options including systemic therapy, local cytoreductive approaches (external beam radiotherapy, cytoreductive radical prostatectomy or minimally-invasive ablative therapy) and metastases-directed therapies (metastasectomy or stereotactic ablative body radiotherapy). All men with newly-diagnosed mHSPC within four months of commencing ADT and WHO performance status 0 to 2 are eligible. Men who have previously consented to a cytoreductive treatment or have developed castrate-resistant disease will be excluded. This study includes a qualitative analysis component, with patients (*n*=15) and healthcare professionals (*n*=5), to identify and define the key attributes associated with treatment options that would warrant trade-off evaluation in a DCE. The main phase component planned recruitment is 300 patients over one year, commencing in January 2021, with planned study completion in March 2022.

Ethics and dissemination: Ethical approval was obtained from the Health Research Authority East of England, Cambridgeshire and Hertfordshire Research Ethics Committee (Reference: 20/EE/0194). Project information will be reported on the publicly available Imperial College London website and the Heath Economics Research Unit (HERU website including the HERU Blog. We will use the social media

For peer review only

For peer review only - http://bmjopen.bmj.com/site/about/guidelines.xhtml

accounts of IP5-MATTER, Imperial Prostate London, HERU and the individual researchers to disseminate key findings following publication. Findings from the study will be presented at national/international conferences and peer-reviewed journals. Authorship policy will follow the recommendations of the International Committee of Medical Journal Editors.

Trial Registration details: NCT04590976

For peer review only

For peer review only - http://bmjopen.bmj.com/site/about/guidelines.xhtml

STRENGTHS AND LIMITATIONS OF THIS STUDY

1. IP5-MATTER will be the first multi-centre discrete choice experiment (DCE) conducted to evaluate men's treatment preferences for local cytoreductive and metastasis-directed therapy in metastatic hormone-sensitive prostate cancer (mHSPC).
2. The study will explore preference heterogeneity according to the patients' personal characteristics.
3. This is the first study to determine if men with mHSPC are willing to accept the potential effect sizes that are reported in randomised studies.
4. It is not feasible to incorporate all factors that may affect patients' treatment preferences (e.g. impact of hospital visits; the impact of drug effectiveness on disease progression).
5. This study will be undertaken in the United Kingdom and may not be generalisable to other men with mHSPC in other countries.

For peer review only

INTRODUCTION

The number of men diagnosed with prostate cancer each year in the United Kingdom (UK) is reported to be 47,000 [1]. In this cohort it is estimated that, 4,500 men will have a diagnosis of *de novo* synchronous metastatic hormone-sensitive prostate cancer (mHSPC) at index presentation [1]. The mainstay of treatment for such men has been hormonal castration with androgen deprivation therapy (ADT) alone. In isolation this treatment modality results in the emergence of a castrate-resistant state within a median of 11 to 18 months that restricts overall survival (OS) to 3.5 years [2, 3].

Recent advances with using ADT in combination with Docetaxel or novel anti-androgens (e.g. Enzalutamide, Abiraterone Acetate, Apalutamide) at diagnosis have led to a rise in the median OS to 4.8 years [3-9]. Current research is considering the role of local cytoreductive approaches (cytoreductive radical prostatectomy, external beam radiotherapy or minimally-invasive ablative therapy) and metastasis-directed interventions (predominantly, surgical metastatectomy or stereotactic ablative body radiotherapy [SABR]) with a view to acquiring further oncological benefit in this setting [1, 10-18].

Research into the role of local cytoreductive and metastasis-directed interventions has, in part, been propagated by the emergence of the principle of an ‘oligo-metastatic’ state [19]. In this context, men are hypothesised to harbour a favourable clinically-defined, low disease burden that lies between ‘locally-advanced’ and ‘poly-metastatic’ disease [19-21]. Early randomised data suggests that men who exhibit such disease may gain the most benefit from additional cytoreductive interventions [10, 22].

These interventions offer potential overall and progression-free survival benefits. Furthermore, they may allow second-line systemic agents and their associated toxicities to be avoided or deferred. However, we currently understand very little about men’s treatment preferences and the decision-making process in this emerging treatment paradigm [23].

The IP5-MATTER study will use a Discrete Choice Experiment (DCE) to determine men’s preferences for, and trade-offs between, the attributes (survival and side-

For peer review only - http://bmjopen.bmj.com/site/about/guidelines.xhtml

effects) of different treatment options for mHSPC including systemic therapy, local cytoreductive and metastases-directed therapies.

DISCRETE CHOICE EXPERIMENT (DCE)

A DCE is a survey method that is related to 'conjoint analysis' [24, 25]. A DCE was selected as the method for this study as it allows the researcher to calculate the trade-offs that patients make between specific treatment options [26]. The trade-off in IP5-MATTER is whether a man is willing to accept the disadvantages to himself (i.e. side effects, risk, visits to hospitals) with an extra treatment(s) in exchange for potential survival or cancer progression-free survival benefits. This is also termed the "marginal rate of substitution" [26].

In the IP5-MATTER study, patients will be presented with hypothetical treatment scenarios [26]. Each scenario will be described using a set of 'attributes' associated with each treatment e.g. treatment modality, urine incontinence, fatigue, loss of erections, length of time until cancer starts to grow again, length of survival after diagnosis. Each treatment scenario will be associated with a realisation or level for each attribute. For instance, in a hypothetical treatment the urine incontinence attribute may take the value of 10% or 1 in 10 men, i.e. of 100 men who have the hypothetical treatment 1 in 10 will experience urine incontinence.

The attributes and levels will be combined into hypothetical treatment scenarios and these will be grouped into treatment choice sets using experimental design theory. In essence, the patient will be asked to 'choose' between two treatments A or B in a choice set.

Each patient will be asked to complete several choice sets each containing different pairs of treatments. Each choice set will vary the levels of the attributes in a systematic way that allows the researcher to identify how the different realisations of the attributes affects the patients' choices (**Figure 1**). We will include up to 12 choices per patient [24, 25].

For peer review only

For peer review only - http://bmjopen.bmj.com/site/about/guidelines.xhtml

PREVIOUS DISCRETE CHOICE EXPERIEMENTS IN PROSTATE CANCER

A number of DCE studies have previously been completed in the field of non-metastatic prostate cancer, metastatic castrate-resistant prostate cancer (mCRPC) and mHSPC suitable to elicit patients' treatment preferences [27-31].

Non-Metastatic Prostate Cancer

Sculpher et al. undertook the first prostate cancer DCE in 129 men in the UK with non-metastatic prostate cancer (T1 or T2) [31]. The primary focus was to evaluate side-effects of various ADT options. The group reported men were willing to forgo up to 3 months of life expectancy to avoid limitations in physical energy [31].

The recent COMPARE (COMparing treatment options for ProstAte cancer) multicentre study focused on 650 men with newly-diagnosed localised non-metastatic prostate cancer [NCT01177865] [32]. Watson and colleagues identified that patients' preferences for attributes for treatment were, in order: survival, no incontinence, not needing further treatment and maintaining an erection. Interestingly, men were willing to trade 0.68% absolute survival for a 1% absolute improvement in urinary function and 0.28% absolute survival for 1% absolute improvement in maintaining erections [32].

Furthermore, King and colleagues in a study of 357 men with localised prostate cancer, who had undergone either radical therapy or commenced ADT, found men were similarly willing to trade-off 6 months of survival if they could avoid erectile dysfunction and approximately 13 months of survival if they could avoid urinary incontinence [28].

Finally, in a study of 110 men with early prostate cancer, as well as their treating urologist, conducted by de Bekker-Grob and colleagues it was reported that whilst urinary incontinence was a concern among both patient and clinician, the risk of erectile dysfunction secondary to radiotherapy was solely valued by the clinician alone [27].

For peer review only - http://bmjopen.bmj.com/site/about/guidelines.xhtml

Metastatic Castrate-Resistant Prostate Cancer

In the mCRPC setting, Eliasson and colleagues study 285 men with mCRPC from three countries (Germany, France and the United Kingdom) [30]. The group reported that lower fatigue (Odds Ratio, 1.365 [95% CI 1.219-1.528]), and fewer additional hospital visits (Odds Ratio, 1.245 [95%CI 1.111-1.397]) were the most important factors affecting any treatment choice [30].

Furthermore, Uemura and colleagues studied 133 Japanese men with mCRPC to demonstrate that fatigue (relative importance [RI] = 24.9 %, [95 % CI: 24.7 - 25.1]) was the most important attribute [33]. This was followed by a reduction in the risk of bone pain (RI = 23.2 %, [95 % CI: 23.0 - 23.5]). The authors' concluded that men were more concerned by quality-of-life secondary to side effects than any potential extension in survival [33].

Metastatic Hormone Sensitive Prostate Cancer

A single DCE in mHSPC has been performed by de Freitas and colleagues on a mixed cohort of 152 men, of which a minority (45/152; 29.6%) had mHSPC. The group evaluated Docetaxel or Abiraterone Acetate in combination with ADT. Within the mHSPC sub-group the primary ranked attribute was drug effectiveness on survival (Relative attribute importance (RAI) = 9.08), followed by pain control (RAI = 6.37). Of note, men in this cohort were least concerned about method of treatment delivery (RAI = 1.51) [29].

In summary, to our knowledge, no studies have undertaken a DCE to elicit men's preferences for systemic therapy in combination with either local cytoreductive and/or metastasis-directed treatments. The aforementioned, DCE studies in prostate cancer (non-metastatic, metastatic castrate-resistant, and metastatic hormone-sensitive prostate cancer) have predominantly highlighted patients' preferences in the context of being offered established systemic therapy options and a select number of bone-targeted agents.

The IP5-MATTER study is a multicentre DCE designed to evaluate novel treatments (cytoreductive prostatectomy, external beam radiotherapy, minimally-invasive ablative therapy and SABR) in addition to systemic therapy for the first time. To appropriately

For peer review only

For peer review only - http://bmjopen.bmj.com/site/about/guidelines.xhtml

invest in emerging cytoreductive prostate and metastasis-directed treatments options that are most acceptable to such patients, we believe that an attempt to formalise our understanding of the trade-offs between oncological benefits and risks in this cohort of patients should be performed.

STUDY HYPOTHESIS

The null hypothesis (*H0*) is that men with *de novo* synchronous hormone-sensitive metastatic prostate cancer will accept the complications and side-effects associated with combined systemic and cytoreductive local prostate and/or metastasis-directed therapy for any potential oncological benefit.

The scientific hypothesis (*H1*) is that men with *de novo* synchronous hormone-sensitive metastatic prostate cancer may not accept the complications and risk of side effects associated with cytoreductive local prostate and/or metastasis directed therapy for any potential oncological benefit.

PATIENT AND PUBLIC INVOLVEMENT

A patient-involvement interview was held with two patients who had metastatic prostate cancer to determine initial patient acceptability of the study. The integrated qualitative component of this study (**Phase 1 & 2**) incorporates patient-involvement. A patient and public involvement (PPI) representative was present during the HRA REC assessment. This representative will continue to be involved throughout the duration of the study. This will include the Trial Management Group (TMG). Furthermore, other patients that are not involved directly in this study will be present on the independent Trials Steering Committee (TSC).

METHODS AND ANALYSIS

STUDY DESIGN

IP5-MATTER will be performed at sites within the United Kingdom (UK). This will be a cross-sectional questionnaire study. The initial qualitative phase (**Phase 1**) will involve semi-structured interviews with (up to 5) clinician members of the multidisciplinary or

For peer review only

tumour board meeting and (up to 5) patients who have had a diagnosis of mHSPC, to identify and define the key attributes associated with treatment options that would warrant trade-off evaluation. These will be used to create the first version of the questionnaire (**Phase 1**) [26].

Interviews with participants will be audio-recorded and transcribed verbatim. These will be destroyed on transcription or within two months (whichever occurs earliest). Transcripts will be analysed using an inductive thematic analysis by at least two researchers. Inductive thematic analysis will be performed as described by Nowell and colleagues 6-step iterative process [34]. Researchers will i) *Familiarize themselves with the data* (e.g. document potential themes/codes) ii) *Generate initial codes* (e.g. reflective journaling) iii) *Search for themes* (e.g. triangulation of data) iv) *Review themes* (e.g. test for referential adequacy to raw data) v) *Define themes and name*, and vi) *Produce a final report* [34]. The responses and suggestions from the interviews will be used to develop the questionnaire in order to ensure that 1) any attribute or levels that do not seem appropriate are removed or modified; 2) any additional attributes or levels that are not in the questionnaire are included; 3) the questionnaire is understandable; and 4) any complex treatment information is explained in a accessible manner [26].

In the DCE questionnaire, the attributes will be combined into different hypothetical scenarios that will be presented to men in the form of a treatment choice. The hypothetical scenarios included in the questionnaire will be selected using experimental design theory. These attributes and levels will be combined to create treatment scenarios and paired into choice sets of two scenarios. We will use NGENE software (ChoiceMetrics) to generate a 36 choice tasks D-efficient design with non-informative (null) priors and allowing estimation of non-linear effects of attributes [35]. The design is based on the main effects only (i.e. without interactions). The 36 choice tasks will be split into three blocks of 12 tasks to reduce respondent burden. Respondents will be allocated to one of the three blocks. Risk attributes will be presented using icon arrays, ratios, and percentages to ease comprehension **(Figure 1)** shows an example choice task. The questionnaire will be trialled on a different group of up to 10 men using a semi-structured 'Think Aloud' interview and any further

For peer review only

For peer review only - http://bmjopen.bmj.com/site/about/guidelines.xhtml

changes made to create the final version of the DCE questionnaire (**Phase 2**) [36]. The final survey instrument will also collect information on patients' characteristics (e,g. age, education).

This final questionnaire will then be used in the main phase of the study in 300 men across a number of UK trial sites over 12 months (**Phase 3**). The DCE questionnaire will be given to the participants to fill in, either directly following a face-to-face hospital clinic appointment or in their own home. For the electronic version of the survey, REDCap software will be used, with tablets available following a face-to-face hospital clinic appointment where required.

For peer review only

STUDY POPULATION

Men diagnosed with *de novo* synchronous metastatic hormone-sensitive prostate cancer who have not consented to a form of local cytoreductive or metastasis-directed therapy.

ELIGIBILITY

INCLUSION CRITERIA

1. Diagnosed with metastatic hormone-sensitive prostate cancer within 4 months of screening visit.
2. WHO performance status 0-2.

EXCLUSION CRITERIA

1. Castrate-resistant metastatic prostate cancer.
2. Patient has consented to a form of local cytoreductive treatment to the prostate.
3. Patient has consented to a form of metastasis-directed therapy.

IDENTIFICATION OF PATIENTS & CONSENT

All men diagnosed with prostate cancer discussed at the multidisciplinary team (MDT) meeting (or a tumour board) as well as any man meeting the eligibility criteria prior to the MDT meeting will be identified for screening. Members of the MDT will identify

patients suitable for IP5-MATTER. The treating clinicians will mention the study and the local Research Nurses, Research Fellows, Clinical Trial Coordinators, Clinical Trial Practitioners or the treating Clinicians will then approach the patient if they are interested.

Patients with confirmed metastatic prostate cancer and who satisfy the entry criteria will be approached for study enrolment. Patients can be included even if they have already commenced ADT, chemotherapy or an alternative hormonal agent as part of their standard of care. They will be provided with a Patient Information Sheet (PIS) and as much time as they might need to consider whether or not they wish to participate (minimum 24 hours).

Patients already aware of their diagnosis can be approached by telephone to enquire as to their interest in the study so that a PIS can be sent out by email or post prior to a clinical visit.

In the qualitative phases (**Phases 1 & 2**), patients who wish to participate after reading the PIS will be given a date for the semi-structure interview (face-to-face or using audio-visual software). In the main **Phase 3** component, patients who meet the eligibility criteria and are willing to participate will be given the questionnaire to fill in their own time either in the face-to-face or virtual hospital clinic or in their own home. Remote informed consent can be undertaken with the assistance of a telephone or audio-visual software, determined by local availability and protocol. An electronic method (in-built REDCap e-consent form mirroring paper version with ‘advanced electronic signature’ using a smartphone, tablet, computer) will enable remote informed consent in accordance with HRA-MHRA e-consent guidance (2018) [37]. Informed consent will be signed before implementation of any study-related procedure.

RANDOMISATION

No randomisation is required for this study.

STUDY OUTCOME MEASURES

For peer review only - http://bmjopen.bmj.com/site/about/guidelines.xhtml

PRIMARY OUTCOMES

- To determine the attributes associated with treatment that are most important to men with metastatic hormone-sensitive prostate cancer.

- To determine men's preferences for, and trade-offs between, the attributes (survival and side-effects) of different treatment options in metastatic prostate cancer including systemic therapy, local cytoreductive and metastases-directed therapies.

SECONDARY OUTCOMES

- To inform treatment pathways that are being developed nationally and internationally following randomised trials that are being conducted to evaluate these questions.

- The effect size from these studies can be used with our IP5-MATTER study to determine if, on average, men are willing to accept the potential effect sizes that are demonstrated in trials.

STUDY VISITS

- **Phase 1 or 2**: Single-visit for semi-structured interview or review of electronic study-developed questionnaire (**Table 1**).

- **Phase 3** (Main Study): Single-visit Completion of the electronic DCE during a routine hospital visit or at patient's own home (**Table 1**).

FOLLOW-UP

There is no planned follow-up for this study.

SAMPLE SIZE CALCULATION AND STATISTICAL ANALYSES

For peer review only - http://bmjopen.bmj.com/site/about/guidelines.xhtml

Sample Size and Power Considerations

IP5-MATTER trial sample size is set at 300. This is based on the premise that the precision of DCE parameter estimates improves until a sample size of 300 and then further gains in precision are small, according to the sampling strategy recommendations set by the International Society for Pharmacoeconomics and Outcomes Research (ISPOR) Good Research Practices for Conjoint Analysis Task Force (2011) [24].

Planned Recruitment Rate

We are aware from the IP2-ATLANTA trial that Centres have approximately 3 men diagnosed with metastatic prostate cancer per month [12, 38]. The initial qualitative phase will focus on the Northwest London regional cancer network (three hospitals) and take 6 weeks to complete. Full phase recruitment is based on 300 patients across 30 sites recruiting a minimum of 10 patients per year or about 1 patient per month. This is an estimated recruitment rate of 26%. Our previous DCEs (i.e. COMPARE) had an acceptance rate of over 50% [32].

Statistical Analysis

Responses from the DCE will be analysed using multinomial logit (MNL) regression techniques that allow for multiple observations from each respondent and preference differences across respondents [26, 32]. This will be used to understand treatment preferences and trade-offs made by patients when considering treatment options of mHSPC. In the MNL analysis, the functional form is specified as:

$$U_{ntj} = \beta X_{ntj} + \varepsilon_{ntj}$$

which represents the utility of option *j* in choice task *t* for patient *n,* where X_{ntj} is a vector of variables representing the levels of the treatment presented in option *j,* β is a vector of utility weights associated with each attribute and ε_{ntj} is the error term. In the analysis, we will assume that patients gain utility (welfare) from treatment and the

For peer review only

amount of welfare depends on the attributes and levels of the treatment. We assume that in each choice set patients choose the treatment that would bring them the highest welfare.

From the results of the DCE, it is possible to estimate the relative importance of attributes and the trade-offs that individuals are willing to make between attributes of a treatment. These trade-offs are how much of one attribute respondents are willing to sacrifice for improvements in another [26].

In this case, we can explore how much life expectancy patients must gain before they are willing to accept treatment side effects. Further, based on the regression results, we can calculate whether the attribute had a positive or negative effect on respondent utility, and the relative importance of a unit change in each attribute when respondents are choosing between treatments. We can also calculate the trade-offs that respondents make between treatment attributes when making choices, which is represented by the ratio of the coefficients [32].

Additionally, we will explore observed preference heterogeneity according to patients' characteristics. The observed characteristics will be analysed by interacting them with the attributes in the MNL model specifications.

DATA COLLECTION

The principal means of data collection from participants will be Electronic Data Capture (EDC) using the web-based REDCap generated platform, provided by the Sponsor. All eCRFs will be completed using de-identified data.

DATA MONITORING & ARCHIVING

On two occasion per year a combined independent data monitoring and trial steering committee will meet. All trial documentation will be archived for a minimum of 10 years following the completion of the study.

TRIAL FUNDING, ORGANISATION AND ADMINISTRATION

For peer review only - http://bmjopen.bmj.com/site/about/guidelines.xhtml

IP5-MATTER was approved by Health Research Authority (HRA) East of England, Cambridgeshire and Hertfordshire Research Ethics Committee (20/EE/0194). The study is a joint collaboration between Imperial College London and Health Economic Research Unit (HERU), University of Aberdeen. This study is funded by the Wellcome Trust (204998/Z/16/Z) & University College London Hospitals (UCLH) Charity (P83624/1348).

The study will be monitored periodically by trial monitors to assess progress and verify adherence to the protocol, ICH GCP E6 guidelines and other national/international requirements and to review the completeness, accuracy and consistency of the data (**Supplementary Material**). The study may be subject to inspection and audit by regulatory bodies to ensure adherence to GCP and the UK Policy Framework for Health and Social Care Research.

DISCUSSION

IP5-MATTER is a multicentre, prospective discrete choice experiment in men with a new diagnosis of *de novo* synchronous mHSPC. This is the first discrete choice experiment, to the author's knowledge, that evaluates men's treatment preferences for local cytoreductive and metastasis-directed therapy following such a diagnosis.

This study will determine men's preferences for, and trade-offs between, the attributes (survival and side-effects) of different treatment options in metastatic prostate cancer including systemic therapy, local cytoreductive and metastases-directed therapies.

When taken collectively, it is expected that the results will be used to determine if, on average, men are willing to accept the potential effect sizes that are reported in current and future randomised studies. This will have wide-reaching implications on the investment in, and provision of, specific treatment options proposed for integration into the current standard of care treatment pathways for men with mHSPC.

Furthermore, it is possible that once such treatment pathways are established the findings from this DCE may be integrated into further work towards the creation of a

For peer review only - http://bmjopen.bmj.com/site/about/guidelines.xhtml

decision treatment aid (DAT) for men with mHSPC. There is residual uncertainty in the role of DCE's in the development of such DATs [39]. However, this methodology is currently being explored in parallel to this study in localised prostate cancer and other benign surgical settings, and if proven IP5-MATTER may offer utility in the development in any future decision treatment aid for this cohort [40, 41].

CONCLUSION

IP5-MATTER answers important questions relating to men's decision-making in relation to local cytoreductive and metastasis-directed treatments following a new diagnosis of metastatic hormone-sensitive prostate cancer.

TRIAL STATUS

IP5-MATTER is opened to recruitment in 30 centres in England, Wales and Scotland in February 2022 and planned recruitment completed by February 2022 and study completion in April 2022.

Acknowledgements: We would like to thank all the participants, study PIs, trial clinicians, research nurses, Imperial Clinical Trial Unit staff and other site staff who have been responsible for setting up, recruiting participants and collecting the data for the IP5-MATTER trial. We are also grateful for the ongoing support of the Trial Management Group and our IP5-MATTER patient representatives. Finally, we would like to thank our trial funder the Wellcome Trust and University College Hospitals London (UCLH) Charity.

Contributors: Conception and design of IP5-MATTER trial: MJC, MG, FHSJ, MW, VW, HUA. All authors have read and approved the final manuscript. MJC, MGG, MG, NS, KTJ, GH, FHJ, NKN, JS, TP, DB, AR, MB, BR, SM, VK, TD, AF, MW, VW, HUA.

Patient consent for publication: Not required.

Conflicts of Interest

Martin J. Connor's research is support by University College London Hospitals (UCLH) Charity and the Wellcome Trust.

For peer review only

For peer review only - http://bmjopen.bmj.com/site/about/guidelines.xhtml

Mesfin Genie and Verity Watson are based at the Health Economics Research Unit (HERU), University of Aberdeen. HERU is funded by the Chief Scientists Office of the Scottish Government Health and Social Care Directorate.

Kamalram Thippu Jayaprakash acknowledges research grant from the UK National Institute of Health Research Clinical Research Network Eastern and has received educational grants from Bayer UK, Janssen Oncology, Pfizer, Roche, and Takeda.

Hashim U. Ahmed's research is supported by core funding from the United Kingdom's National Institute of Health Research (NIHR) Imperial Biomedical Research Centre. Ahmed currently receives funding from the Wellcome Trust, Prostate Cancer UK, MRC (UK), Cancer Research UK, The Urology Foundation, BMA Foundation, Imperial Healthcare Charity, Sonacare Inc., Trod Medical, and Sophiris Biocorp for trials in prostate cancer. Ahmed was a paid medical consultant for Sophiris Biocorp, Sonacare Inc. and BTG in the past 3 years. He is currently a paid proctor for Sonacare and Boston.

All other authors have no declaration

References

1 Connor MJ, Shah TT, Horan G, et al. Cytoreductive treatment strategies for de novo metastatic prostate cancer, *Nature Reviews Clinical Oncology* 2019:1-15.

2 Miyake H, Matsushita Y, Watanabe H, et al. Prognostic significance of time to castration resistance in patients with metastatic castration-sensitive prostate cancer, *Anticancer Res* 2019;39:1391-6.

3 Marchioni M, Di Nicola M, Primiceri G, et al. New antiandrogen compounds compared to docetaxel for metastatic hormone sensitive prostate cancer: results from a network meta-analysis, *J Urol* 2020;203:751-9.

4 Sweeney CJ, Chen Y, Carducci M, et al. Chemohormonal therapy in metastatic hormone-sensitive prostate cancer, *N Engl J Med* 2015;373:737-46.

5 Fizazi K, Tran N, Fein L, et al. Abiraterone plus prednisone in metastatic, castration-sensitive prostate cancer, *N Engl J Med* 2017;377:352-60.

For peer review only - http://bmjopen.bmj.com/site/about/guidelines.xhtml

6 James ND, de Bono JS, Spears MR, et al. Abiraterone for prostate cancer not previously treated with hormone therapy, *N Engl J Med* 2017;377:338-51.

7 Chi KN, Agarwal N, Bjartell A, et al. Apalutamide for metastatic, castration-sensitive prostate cancer, *N Engl J Med* 2019;381:13-24.

8 James ND, Sydes MR, Clarke NW, et al. Addition of docetaxel, zoledronic acid, or both to first-line long-term hormone therapy in prostate cancer (STAMPEDE): survival results from an adaptive, multiarm, multistage, platform randomised controlled trial, *Lancet* 2016;387:1163-77 doi:10.1016/S0140-6736(15)01037-5 [doi] [published Online First: March 19].

9 James ND, Spears MR, Clarke NW, et al. Survival with newly diagnosed metastatic prostate cancer in the "docetaxel era": data from 917 patients in the control arm of the STAMPEDE trial (MRC PR08, CRUK/06/019), *Eur Urol* 2015;67:1028-38.

10 Connor MJ, Smith A, Miah S, et al. Targeting Oligometastasis with Stereotactic Ablative Radiation Therapy or Surgery in Metastatic Hormone-sensitive Prostate Cancer: A Systematic Review of Prospective Clinical Trials, *European Urology Oncology* 2020.

11 De Bruycker A, Tran PT, Achtman AH, et al. Clinical perspectives from ongoing trials in oligometastatic or oligorecurrent prostate cancer: an analysis of clinical trials registries, *World J Urol* 2020:1-10.

12 ClinicalTrials.gov [Internet]. Additional Treatments to the Local Tumour for Metastatic Prostate Cancer: Assessment of Novel Treatment Algorithms (IP2-ATLANTA). NCT03763253. . 2018;2020.

13 ClinicalTrials.gov [Internet]. Androgen Deprivation Therapy or Androgen Deprivation Therapy Plus Definitive Treatment (Radiation or Surgery) FUSCC-OMPCa. NCT02742675. 2016;2020.

14 ClinicalTrials.gov [Internet]. Best Systemic Therapy or Best Systemic Therapy (BST) Plus Definitive Treatment (Radiation or Surgery). NCT01751438. 2012;2020.

15 ClinicalTrials.gov [Internet]. Standard Systemic Therapy With or Without Definitive Treatment in Treating Participants With Metastatic Prostate Cancer (SWOG 1802). NCT03678025. . 2018;2020.

16 ClinicalTrials.gov [Internet]. Impact of Radical Prostatectomy as Primary Treatment in Patients With Prostate Cancer With Limited Bone Metastases (g-RAMPP). NCT02454543. 2015;2020.

17 ClinicalTrials.gov [Internet]. Cytoreductive Prostatectomy Versus Cytoreductive Prostate Irradiation as a Local Treatment Option for Metastatic Prostate Cancer: a Multicentric Feasibility Trial (LoMP II). NCT03655886. 2018;2020.

18 ClinicalTrials.gov [Internet]. Testing Radical Prostatectomy in Chinese Men With Prostate Cancer and oligoMetastases to the Bone. NCT03988686. 2019;2020.

For peer review only

For peer review only - http://bmjopen.bmj.com/site/about/guidelines.xhtml

19 Weichselbaum RR, Hellman S. Oligometastases revisited, *Nature reviews Clinical oncology* 2011;8:378.

20 Tosoian JJ, Gorin MA, Ross AE, et al. Oligometastatic prostate cancer: definitions, clinical outcomes, and treatment considerations, *Nature Reviews Urology* 2017;14:15.

21 Khoo V. New concepts in prostate cancer management: the conundrum of managing oligometastatic disease in prostate cancer—through the looking glass darkly, *Clin Radiol* 2019.

22 Parker CC, James ND, Brawley CD, et al. Radiotherapy to the primary tumour for newly diagnosed, metastatic prostate cancer (STAMPEDE): a randomised controlled phase 3 trial, *Lancet* 2018;392:2353-66 doi:S0140-6736(18)32486-3 [pii] [published Online First: December 01].

23 Connor MJ, Khoo V, Watson V, et al. Radical Treatment Without Cure: Decision-making in Oligometastatic Prostate Cancer, *Eur Urol* 2021:S0302-3.

24 Bridges JF, Hauber AB, Marshall D, et al. Conjoint analysis applications in health—a checklist: a report of the ISPOR Good Research Practices for Conjoint Analysis Task Force, *Value in health* 2011;14:403-13.

25 Johnson FR, Lancsar E, Marshall D, et al. Constructing experimental designs for discrete-choice experiments: report of the ISPOR conjoint analysis experimental design good research practices task force, *Value in health* 2013;16:3-13.

26 Lancsar E, Louviere J. Conducting discrete choice experiments to inform healthcare decision making, *Pharmacoeconomics* 2008;26:661-77.

27 de Bekker-Grob EW, Bliemer M, Donkers B, et al. Patients' and urologists' preferences for prostate cancer treatment: a discrete choice experiment, *Br J Cancer* 2013;109:633-40.

28 King MT, Viney R, Smith DP, et al. Survival gains needed to offset persistent adverse treatment effects in localised prostate cancer, *Br J Cancer* 2012;106:638-45.

29 de Freitas HM, Ito T, Hadi M, et al. Patient Preferences for Metastatic Hormone-Sensitive Prostate Cancer Treatments: A Discrete Choice Experiment Among Men in Three European Countries, *Adv Ther* 2019;36:318-32.

30 Eliasson L, de Freitas HM, Dearden L, et al. Patients' preferences for the treatment of metastatic castrate-resistant prostate cancer: A discrete choice experiment, *Clin Ther* 2017;39:723-37.

31 Sculpher M, Bryan S, Fry P, et al. Patients' preferences for the management of non-metastatic prostate cancer: discrete choice experiment, *BMJ* 2004;328:382.

32 Watson V, McCartan N, Krucien N, et al. Evaluating the Trade-offs Men with Localized Prostate Cancer Make between the Risks and Benefits of Treatments: The COMPARE Study, *J Urol* 2020:10.1097/JU. 0000000000000754.

For peer review only - http://bmjopen.bmj.com/site/about/guidelines.xhtml

645 33 Uemura H, Matsubara N, Kimura G, et al. Patient preferences for treatment of castration-resistant prostate cancer in Japan: a discrete-choice experiment, *BMC urology* 2016;16:63.

648 34 Nowell LS, Norris JM, White DE, et al. Thematic analysis: Striving to meet the trustworthiness criteria, *International journal of qualitative methods* 2017;16:1609406917733847.

651 35 Rose JM, Bliemer MC. Sample size requirements for stated choice experiments, *Transportation* 2013;40:1021-41.

653 36 Eccles DW, Arsal G. The think aloud method: what is it and how do I use it? *Qualitative Research in Sport, Exercise and Health* 2017;9:514-31.

655 37 Health Research Authority. Joint statement on seeking consent by electronic methods. *Health Research Authority (HRA)* 2018.

657 38 Connor MJ, Shah TT, Smigielska K, et al. Additional Treatments to the Local tumour for metastatic prostate cancer-Assessment of Novel Treatment Algorithms (IP2-ATLANTA): protocol for a multicentre, phase II randomised controlled trial, *BMJ open* 2021;11:e042953.

661 39 Genie MG, Nicoló A, Pasini G. The role of heterogeneity of patients' preferences in kidney transplantation, *J Health Econ* 2020;72:102331.

663 40 Dowsey MM, Scott A, Nelson EA, et al. Using discrete choice experiments as a decision aid in total knee arthroplasty: study protocol for a randomised controlled trial, *Trials* 2016;17:1-10.

666 41 National Cancer Research Institute, (NCRI). NCRI Prostate Group Annual Report 2019-20. [Putting men's preferences at the center of the doctor-patient relationship: The Prostate cAnceR TreatmeNt prEfeRences (PARTNER) Test]. 2020. *Available at:* https://www.ncri.org.uk/wp-content/uploads/NCRI-Prostate-Group-Annual-Report-2019-20.pdf

671

672

673

For peer review only - http://bmjopen.bmj.com/site/about/guidelines.xhtml

Table and Figure Legends

Table 1. Visit Schedule. *Legend: This study does not have a follow-up visit.*

Figure 1. Example choice task. *Legend: Example choice task for this study that is subject to change following the completion of Phase I and Phase II.*

For peer review only

For peer review only - http://bmjopen.bmj.com/site/about/guidelines.xhtml

Table 1. Visit Schedule

IP5-MATTER		
**Visit**	**Screening**	**Visit 1**
**Week**	**0**	**0**
**Informed Consent**	X	
**Inclusion & Exclusion**	X	
**Demographics**	X	
**Medical History**	X	
**Study Intervention: Discrete Choice Experiment**		X

Legend: This study does not have a follow-up visit.

For peer review only

TREATMENT FEATURES	Treatment A	Treatment B
How your metastatic prostate cancer is managed	Hormonal therapies (with or without chemotherapy)<br><br>followed by **no additional treatment** to prostate	Hormonal therapies (with or without chemotherapy)<br><br>followed by **an overnight-stay surgery** and **4 weeks** of recovery time
Is specialised radiotherapy to cancer deposits included?	No	Yes, there are hospital appointments every day for one week to receive this specialist radiotherapy.
How long a man, on average, is expected to live after the diagnosis?	50 months	70 months
How long, on average, until the cancer starts to grow again?	40 months	60 months
Proportion of men who have permanent urinary incontinence after treatment (%)	5% (5 out of 100)<br>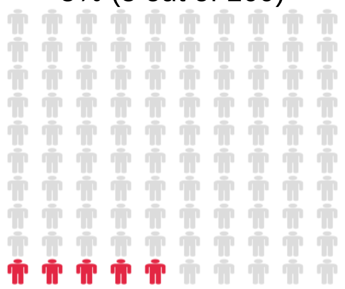	20% (20 out of 100)<br>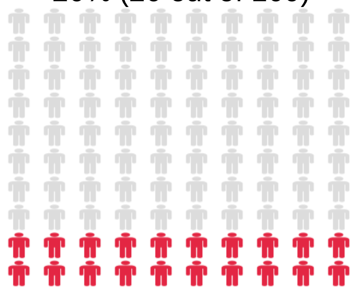
The proportion of men who are not able to maintain an erection sufficient for intercourse (%)	40% (40 out of 100)<br>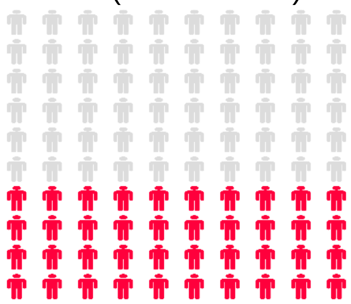	70% (70 out of 100)<br>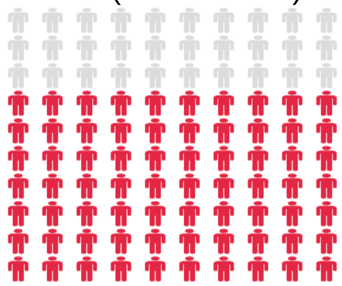
The proportion of men who have a feeling of extreme tiredness (fatigue) impacting on daily activities (%)	10% (10 out of 100)<br>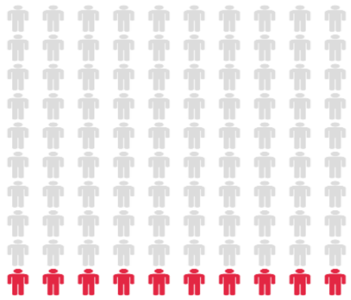	40% (40 out of 100)<br>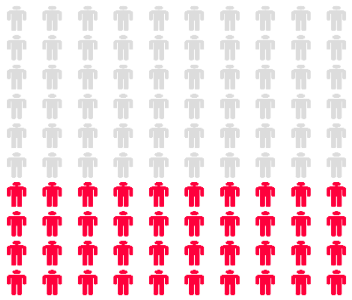
**Which treatment option would you choose?**	☐	☐

SUPPLEMENTARY MATERIAL

1. FULL STUDY DETAILS

Full Study Title: Patients' preferences in the treatment of hormone-sensitive metastatic prostate cancer: a discrete choice experiment

Short Study title / Acronym: Metastatic prostate cancer men's Attitudes towards Treatment of the local Tumour and metastasis Evaluative Research (**IP5-MATTER**)

Product: Discrete Choice Experiment

Development Phase: Multicentre Observational Study

Sponsor: Imperial College London

Version no: 2.0

Protocol Date: 27th August 2020

**IRAS ID:**	276834
**REC Reference Number:**	20/EE/0194
**Clinical trials.gov Number:**	NCT04590976
**Sponsor Protocol Number:**	20CX5760
**Funder reference:**	Wellcome Trust. Senior Clinical Fellowship. Ref: 204998/Z/16/Z. University College Hospitals London (UCLH) Charity (P83624/1348)

2. REGULATORY, ETHICAL AND LEGAL ISSUES

2.1 Declaration of Helsinki

The investigator will ensure that this study is conducted in full conformity with the 7th revision of the 1964 Declaration of Helsinki.

2.2 Good Clinical Practice

The study will be conducted in accordance with the guidelines laid down by the International Conference on Harmonisation for Good Clinical Practice (ICH GCP E6 guidelines).

2.3 Independent Ethics Committee Approval

(i) Initial Approval

Prior to the enrolment of subjects, the REC must provide written approval of the conduct of the study at named sites, the protocol and any amendments, the Patient Information Sheet and Consent Form, any other written information that will be provided to the subjects, any advertisements that will be used and details of any subject compensation.

For peer review only

(ii) Approval of Amendments

Proposed amendments to the protocol and aforementioned documents must be submitted to the REC for approval as instructed by the Sponsor. Amendments requiring REC approval may be implemented only after a copy of the REC's approval letter has been obtained.

Amendments that are intended to eliminate an apparent immediate hazard to subjects may be implemented prior to receiving Sponsor or REC approval. However, in this case, approval must be obtained as soon as possible after implementation.

(iii) Annual Progress Reports

The REC will be sent annual progress reports in accordance with national requirements.

(iv) Annual Progress Reports and End of Trial Notification

The REC will be sent annual progress updates in order to facilitate their continuing review of the study (reference. ICH GCP E6 Section 3.1.4) and will also be informed about the end of the trial, within the required timelines. The Annual Progress Report will detail all SAEs recorded.

2.4 HRA approval

Health Research Authority (HRA) approval will be obtained prior to starting the study. Each participating site will confirm capacity and capability prior to commencing.
The HRA and all participating sites also need to be notified of all protocol amendments to assess whether the amendment affects the institutional approval for each site.

2.5 Other Required Approvals

None

2.6 Non-Compliance and Serious Breaches

All protocol deviations and protocol violations will be reported via MATTER trial team email address and reviewed by the Principal Investigator and reported to the trial TMG on a monthly basis. Protocol violations will be reported to the Sponsor.

An assessment of whether the protocol deviation/violation constitutes a serious breach will be made.

A serious breach is defined as:

A breach of the conditions and principles of GCP in connection with a trial or the trial protocol, which is likely to affect to a significant degree:

- The safety or physical or mental integrity of the UK trial subjects; or
- The overall scientific value of the trial

For peer review only

The Sponsor will be notified within 24 hours of identifying a likely Serious Breach. If a decision is made that the incident constitutes a Serious Breach, this will be reported to the REC within 7 days of becoming aware of the serious breach.

2.7 Insurance and Indemnity

Imperial College London holds negligent harm and non-negligent harm insurance policies, which apply to this study. *Imperial College London will act as the main Sponsor for this study. Delegated responsibilities will be assigned to the participating sites taking part in this study.*

2.8 Trial Registration

The study will be registered on a trial database e.g. ClinicalTrials.gov in accordance with requirements of the International Committee of Medical Journal Editors (ICMJE) regulations.

2.9 Informed Consent

Subjects will be provided with a copy of the signed Subject Information Sheet/Informed Consent Form document. The original Informed Consent Form should be retained with the source documents.

2.10 Contact with General Practitioner

It is the investigator's responsibility to inform the subject's General Practitioner (where applicable) by letter that the subject is taking part in the study provided the subject agrees to this, and information to this effect is included in the Subject Information Sheet and Informed Consent. A copy of the letter should be filed in the medical notes.

2.11 Subject Confidentiality

The investigator must ensure that the subject's confidentiality is maintained. On the CRF or other documents submitted to the Sponsors, subjects will be identified by a subject ID number only. Documents that are not submitted to the Sponsor (e.g., signed informed consent form) should be kept in a strictly confidential file by the investigator.

The investigator shall permit direct access to subjects' records and source documents for the purposes of monitoring, auditing, or inspection by the Sponsor, authorised representatives of the Sponsor and RECs.

2.12 Data Protection and Patient Confidentiality

The investigator will preserve the confidentiality of all participants taking part in the study, which will be conducted in accordance with GDPR and the Data Protection Act. Furthermore, all investigators and study staff must comply with the requirements of the Data Protection Act 2018 with regards to the collection, storage, processing and disclosure of personal information and will uphold the Act's core principles.

2.13 End of Trial

The end of the trial will be marked by the completion of the study report so that all study outcomes can be addressed. However, the trial may end early upon recommendation of the Trial Steering Committee.

For peer review only

2.14 Study Documentation and Data Storage

The study investigators and study site staff will comply with the requirements of the Data Protection Act 2018 concerning the collection, storage, processing and disclosure of personal information and will uphold the Act's core principles.

The investigator must retain essential documents until notified by the Sponsor, and for at least ten years after study completion. Subject files and other source data (including copies of protocols, CRFs, original reports of test results, correspondence, records of informed consent, and other documents pertaining to the conduct of the study) must be retained. Documents should be stored in such a way that they can be accessed/data retrieved at a later date.

All research documentation and information will undergo a process of pseudonymisation where possible. Whilst within the study, patients will be identified by a unique study number, and the data in the CRF will be linked to this number. Research data will be entered onto a dedicated, secure, encrypted trial database, specifically constructed for this purpose. The study team will maintain the confidentiality of all patient data and will not disclose information by which patients may be identified to any third party other than those directly involved in the treatment of the patient and organisations for which the patient has given explicit consent for data transfer. Data within the NHS system such as patient notes, and reports will remain confidential in accordance with NHS confidentiality code of practice.

Paper enrolment logs, including patients' names, NHS numbers and dates of birth, will be kept in the Investigator Site File and will be stored in a secure, locked room within each participating site. Electronic documents will be kept on secure and encrypted computers. Access to these documents will be highly restricted and will only be available to the relevant research team members.

No study documents will be destroyed without prior written agreement between the Sponsor and the investigator. Should the investigator wish to assign the study records to another party or move them to another location, written agreement must be obtained from the Sponsor.

However, psuedonymised data will be shared with Dr Verity Watson at the Health Economics Research Unit (HERU), University of Aberdeen, Aberdeen for analysis at completion of the study.

3. STUDY WITHDRAWAL

3.1 Permanent Discontinuation of Study Treatment and Withdrawal from Study

(v) Permanent discontinuation of study treatment

Subjects may discontinue study treatment for the following reasons:

- At the request of the subject.
- Adverse event/ Serious Adverse Event
- If the investigator considers that a subject's health will be compromised due to adverse events or concomitant illness that develop after entering the study.

(vi) Withdrawal from Study

Withdrawal from the study refers to discontinuation of study treatment and study procedures and can occur for the following reasons:

- Subject decision

- Loss to follow-up

3.2 Adverse Events

DEFINITIONS

Adverse Event (AE): any untoward medical occurrence in a patient or clinical study subject.

Serious Adverse Event (SAE): any untoward and unexpected medical occurrence or effect that:

- **Results in death**
- **Is life-threatening** – *refers to an event in which the subject was at risk of death at the time of the event; it does not refer to an event which hypothetically might have caused death if it were more severe*
- **Requires hospitalisation, or prolongation of existing inpatients' hospitalisation**
- **Results in persistent or significant disability or incapacity**
- **Is a congenital anomaly or birth defect**

Medical judgement should be exercised in deciding whether an AE is serious in other situations. Important AEs that are not immediately life-threatening or do not result in death or hospitalisation but may jeopardise the subject or may require intervention to prevent one of the other outcomes listed in the definition above, should also be considered serious.

REPORTING PROCEDURES

All adverse events should be reported. Depending on the nature of the event the reporting procedures below should be followed. Any questions concerning adverse event reporting should be directed to the Chief Investigator in the first instance.

Non serious AEs
All such events, whether expected or not, should be recorded.

Serious AEs
An SAE form should be completed on RedCap and also an email sent to the Chief Investigator within 24 hours. However, relapse and death due to metastatic prostate cancer, and hospitalisations for elective treatment of a pre-existing condition do not need reporting as SAEs.

All SAEs should be reported to the **HRA East of England, Cambridgeshire and Hertfordshire Research Ethics Committee (20/EE/0194)** where in the opinion of the Chief Investigator, the event was:

- 'related', ie resulted from the administration of any of the research procedures; and
- 'unexpected', ie an event that is not listed in the protocol as an expected occurrence

Reports of related and unexpected SAEs should be submitted within 15 days of the Chief Investigator becoming aware of the event, using the NRES SAE form for non-IMP studies. The Chief Investigator must also notify the Sponsor of all SAEs.

Local investigators should report any SAEs as required by their Local Research Ethics Committee, Sponsor and/or Research & Development Office

For peer review only

4. DATA MANAGEMENT

4.1 Source Data

Source documentation is defined as the first-time data appears, and may include original document, data and records (hospital records, clinical reports, MRI and Ultrasound reports, other procedure reports, laboratory notes, other data recorded at the pathology and biochemistry laboratories, etc.). Information in source documents (e.g. medical history) dated prior to the Informed Consent Form signature date may be used to verify patient suitability for the study.

Clinical records must be marked to indicate a subject has been enrolled into the clinical study.

The Investigator must ensure the availability of source documents from which the information on the eCRF was obtained. Where printouts and electronic medical records are provided as source documents, they should be signed and dated by a member of the adequately trained research team, to indicate that the data provided is a true reproduction of the original source document.

All study data may be inspected by the Sponsor and regulatory authorities by people working on behalf of the Sponsor, and by representatives of Regulatory Authorities, where it is relevant to this research.

4.2 Language

CRFs will be in English. Generic names for concomitant medications should be recorded in the CRF wherever possible. All written material to be used by subjects must use vocabulary that is clearly understood and be in the language appropriate for the study site.

4.3 Database

Data collection will be via Electronic Data Capture (EDC) using the online REDCap database. Data is entered into the EDC system via site personnel. All source data will be recorded in the CRF and the completed case report book will be signed by the Investigator or his/her appropriate designee. All changes made at any time following the electronic signing will have an electronic audit trail with a signature and date. The completed case report book will then again be signed by the investigator or his/her appropriate designee. Specific instructions and further details will be outlined in the CRF manual.

4.4 Data Collection

All study data will be entered into electronic Case Report (eCRFs) in a database provided by using REDCap. All eCRFs will be completed using de-identified data.

CRF completion may be delegated by the Principal Investigator (documented on the Delegated Task List) to other study personnel though the Principal Investigator remains responsible for the accuracy and integrity of all data entered to eCRFs. Further details of procedures for CRF/eCRF completion, including data review, database cleaning, issuing and resolving data queries, and identification of steps for creation, modification, maintenance and

For peer review only

archiving of source data via any computerised systems will be provided in the study specific Data Management Plan (CRF manual).

4.5 Archiving

All trial documentation, including that held at participating sites and the trial coordinating centre, will be archived for a minimum of 10 years following the end of the study. Subject files and other source data (including copies of protocols, CRFs, original reports of test results, correspondence, records of informed consent, and other documents pertaining to the conduct of the study) must be retained. Documents should be stored in such a way that they can be accessed/data retrieved later. Consideration should be given to security and environmental risks.

No study document will be destroyed without prior written agreement between the Sponsor and the investigator. Should the investigator wish to assign the study records to another party or move them to another location, written agreement must be obtained from the Sponsor.

Storage and handling of confidential trial data and documents will be in accordance with the Data Protection Act 2018 (UK).

5. STUDY MANAGEMENT STRUCTURE

5.1 Trial Steering Committee

A Trial Steering Committee (TSC) will be convened including as a minimum an Independent Chair, Independent Clinician, the Chief Investigator and Study Manager. The role of the TSC is to provide overall supervision of trial conduct and progress. Details of membership, responsibilities and frequency of meetings will be defined in a separate Charter.

5.2 Trial Management Group

A Trial Management Group (TMG) will be convened including the Principal Investigators, co-investigators and key collaborators, Study Statistician and Study Manager. The TMG will be responsible for day-to-day conduct of the trial and operational issues. Details of membership, responsibilities and frequency of meetings will be defined in separate Terms of Reference. Authorship of publications will be determined by the TMG. When necessary, decisions will be referred to the TSC. Meetings will be scheduled in a risk-adapted manner to allow for the review of events during the trial.

5.3 Data Monitoring Committee (DMC)

A combined data monitoring and trial steering committee will meet twice a year basis. The composition of this committee will include but not be limited to the Chief Investigator, Trial Statistician, Trial Coordinator, Research nurse and Patient representative.

5.4 Early Discontinuation of the Study

The following reasons may result in early discontinuation:

- It is not feasible to reach the planned outcomes (e.g. recruitment rate will not reach 300).

The statistical criteria for termination of the study will be detailed in the statistical analysis plan (SAP).

5.5 Risk Assessment

A study-specific risk assessment will be performed prior to the start of the study to assign a risk category of 'low', 'medium' or 'high' to the trial. Risk assessment will be carried out by Prof Hashim Ahmed and Mr Martin Connor in collaboration with the TMG the result will be used to guide a monitoring plan. The risk assessment will consider all aspects of the study and will be updated as required during the course of the study.

5.6 Monitoring

The study will be monitored periodically by trial monitors to assess the progress of the study, verify adherence to the protocol, ICH GCP E6 guidelines and other national/international requirements and to review the completeness, accuracy and consistency of the data.

Monitoring procedures and requirements will be documented in a Monitoring Plan, developed in accordance with the risk assessment.

5.7 Quality Control and Quality Assurance

Quality Control will be performed according to internal procedures. The study may be audited by a Quality Assurance representative of the Sponsor. All necessary data and documents will be made available for inspection.
The study may be subject to inspection and audit by regulatory bodies to ensure adherence to GCP and the UK Policy Framework for Health and Social Care Research.

5.8 Peer review

This study has been peer reviewed by The Urology Foundation (TUF), the Royal College of Surgeons of England and the British Medical Association (Appendix A). The study is funded by UCLH Charity, 5th Floor East, 250 Euston Road London, NW1 2PG (Appendix B). It has been reviewed within the Urology Department at Imperial College London and the Health Economics Research Unit, Polwarth Building, University of Aberdeen.

5.9 Patient and Public Involvement

5.10 Publication and Dissemination policy

Information concerning the study, patent applications, processes, scientific data or other pertinent information is confidential and remains the property of the Sponsor. The investigator may use this information for the purposes of the study only.

It is understood by the investigator that the Sponsor will use information developed in this clinical study in connection with the development of the ablative or radiotherapy or surgical techniques and, therefore, may disclose it as required to other clinical investigators and to Regulatory Authorities. In order to allow the use of the information derived from this clinical study, the investigator understands that he/she has an obligation to provide complete test results and all data developed during this study to the Sponsor.

Verbal or written discussion of results prior to study completion and full reporting should only be undertaken with written consent from the Sponsor.

Therefore, all information obtained as a result of the study will be regarded as CONFIDENTIAL, at least until appropriate analysis and review by the investigator(s) are completed.

For peer review only

Permission from the Executive/Writing Committee is necessary prior to disclosing any information relative to this study outside of the Trial Steering Committee. Any request by site investigators or other collaborators to access the study dataset must be formally reviewed by the TMG.

A Clinical Study Report summarising the study results will be prepared and submitted to the REC within a year of the end of study.

For peer review only

6. INFORMED CONSENT FORM

INFORMED CONSENT FORM (Phase 3/Main Study)

IP5-MATTER

Metastatic prostate cancer men's **A**ttitudes towards **T**reatment of the local **T**umour and metastasis **E**valuative **R**esearch (**IP5-MATTER**)

Chief Investigator: Professor Hashim U. Ahmed

Please initial each box below. Do not tick

1	I confirm that I have read and understand the patient information sheet dated _ _ / _ _ / _ _ _ _ (Version ___) for the **MATTER** Phase 3 / Main Study. I have had the opportunity to consider the information, ask questions and have had these answered satisfactorily.	
2	I understand that my participation is voluntary and that I am free to withdraw at any time, without giving reason and without my medical care or legal rights being affected.	
3	I understand that relevant sections of my medical notes and data collected during the study, may be looked at by individuals from the Sponsor of the trial (Imperial College London) and responsible persons authorised by the Sponsor, from regulatory authorities or from the NHS Trust, where it is relevant to my taking part in this research. I give permission for these individuals to have access to my records.	
4	I understand that I can stop the questionnaire at any time, for any reason.	
5	I understand that the information collected about me will be used to support other ethically approved research in future, and may be shared anonymously with other researchers.	
6	I give permission for the researchers to contact me regarding this trial during this trial period.	
7	I agree for my GP and other doctors to be informed of my participation in this study and of any clinical relevant study results.	
8	I agree to take part in the above study.	

All the boxes above must be initialled for consent to be valid

Site Number: INSERT
Protocol Number: 20CX5760

Version 2.0 27 AUG 2020
IRAS Number: 276834

For peer review only

OPTIONAL CONSENT

Please initial each box below. Do not tick

10	I give consent for information collected about me to be used to support other ethically approved research in the future, including those outside of the EEA	
11	I give consent to being contacted to potentially taking part in other ethically approved research studies.	

Name of Participant Signature Date

Name of Person taking consent Signature Date

Please give one copy of the consent form to the patient, file one copy in the patient's medical records, and retain the original in the Investigator Site File

For peer review only

For peer review only - http://bmjopen.bmj.com/site/about/guidelines.xhtml

STANDARD PROTOCOL ITEMS: RECOMMENDATIONS FOR INTERVENTIONAL TRIALS

SPIRIT 2013 Checklist: Recommended items to address in a clinical trial protocol and related documents*

Page No.	Section/item	Item No	Description
	**Administrative information**		
**1**	Title	1	Descriptive title identifying the study design, population, interventions, and, if applicable, trial acronym
**2**	Trial registration	2a	Trial identifier and registry name. If not yet registered, name of intended registry
**2,3**		2b	All items from the World Health Organization Trial Registration Data Set
**Suppl.**	Protocol version	3	Date and version identifier
**3,14, 15**	Funding	4	Sources and types of financial, material, and other support
**1,2,16**	Roles and responsibilities	5a	Names, affiliations, and roles of protocol contributors
**14,15**		5b	Name and contact information for the trial sponsor
**14,15, Suppl.**		5c	Role of study sponsor and funders, if any, in study design; collection, management, analysis, and interpretation of data; writing of the report; and the decision to submit the report for publication, including whether they will have ultimate authority over any of these activities
**Suppl.**		5d	Composition, roles, and responsibilities of the coordinating centre, steering committee, endpoint adjudication committee, data management team, and other individuals or groups overseeing the trial, if applicable (see Item 21a for data monitoring committee)

For peer review only

For peer review only - http://bmjopen.bmj.com/site/about/guidelines.xhtml

	**Introduction**		
**5-8**	Background and rationale	6a	Description of research question and justification for undertaking the trial, including summary of relevant studies (published and unpublished) examining benefits and harms for each intervention
**5-8**		6b	Explanation for choice of comparators
**8**	Objectives	7	Specific objectives or hypotheses
**9,10**	Trial design	8	Description of trial design including type of trial (eg, parallel group, crossover, factorial, single group), allocation ratio, and framework (eg, superiority, equivalence, noninferiority, exploratory)
	**Methods: Participants, interventions, and outcomes**		
**10-11**	Study setting	9	Description of study settings (eg, community clinic, academic hospital) and list of countries where data will be collected. Reference to where list of study sites can be obtained
**10-11**	Eligibility criteria	10	Inclusion and exclusion criteria for participants. If applicable, eligibility criteria for study centres and individuals who will perform the interventions (eg, surgeons, psychotherapists)
**9**	Interventions	11a	Interventions for each group with sufficient detail to allow replication, including how and when they will be administered
**Suppl.**		11b	Criteria for discontinuing or modifying allocated interventions for a given trial participant (eg, drug dose change in response to harms, participant request, or improving/worsening disease)
**11**		11c	Strategies to improve adherence to intervention protocols, and any procedures for monitoring adherence (eg, drug tablet return, laboratory tests)
**10**		11d	Relevant concomitant care and interventions that are permitted or prohibited during the trial
**11-12**	Outcomes	12	Primary, secondary, and other outcomes, including the specific measurement variable (eg, systolic blood pressure), analysis metric (eg, change from baseline, final value, time to event), method of aggregation (eg, median, proportion), and time point for each outcome. Explanation of the clinical relevance of chosen efficacy and harm outcomes is strongly recommended

For peer review only - http://bmjopen.bmj.com/site/about/guidelines.xhtml

**12, Table 1**	Participant timeline	13	Time schedule of enrolment, interventions (including any run-ins and washouts), assessments, and visits for participants. A schematic diagram is highly recommended (see Figure)
**3, 12-13**	Sample size	14	Estimated number of participants needed to achieve study objectives and how it was determined, including clinical and statistical assumptions supporting any sample size calculations
**10-11**	Recruitment	15	Strategies for achieving adequate participant enrolment to reach target sample size
	**Methods: Assignment of interventions (for controlled trials)**		
	Allocation:		
**N/A**	Sequence generation	16a	Method of generating the allocation sequence (eg, computer-generated random numbers), and list of any factors for stratification. To reduce predictability of a random sequence, details of any planned restriction (eg, blocking) should be provided in a separate document that is unavailable to those who enrol participants or assign interventions
**N/A**	Allocation concealment mechanism	16b	Mechanism of implementing the allocation sequence (eg, central telephone; sequentially numbered, opaque, sealed envelopes), describing any steps to conceal the sequence until interventions are assigned
**N/A**	Implementation	16c	Who will generate the allocation sequence, who will enrol participants, and who will assign participants to interventions
**N/A**	Blinding (masking)	17a	Who will be blinded after assignment to interventions (eg, trial participants, care providers, outcome assessors, data analysts), and how
**N/A**		17b	If blinded, circumstances under which unblinding is permissible, and procedure for revealing a participant's allocated intervention during the trial

	**Methods: Data collection, management, and analysis**		
**18**	Data collection methods	18a	Plans for assessment and collection of outcome, baseline, and other trial data, including any related processes to promote data quality (eg, duplicate measurements, training of assessors) and a description of study instruments (eg, questionnaires, laboratory tests) along with their reliability and validity, if known. Reference to where data collection forms can be found, if not in the protocol
**18**		18b	Plans to promote participant retention and complete follow-up, including list of any outcome data to be collected for participants who discontinue or deviate from intervention protocols
**18, Suppl.**	Data management	19	Plans for data entry, coding, security, and storage, including any related processes to promote data quality (eg, double data entry; range checks for data values). Reference to where details of data management procedures can be found, if not in the protocol
**12-13**	Statistical methods	20a	Statistical methods for analysing primary and secondary outcomes. Reference to where other details of the statistical analysis plan can be found, if not in the protocol
**12-13**		20b	Methods for any additional analyses (eg, subgroup and adjusted analyses)
**12, Suppl.**		20c	Definition of analysis population relating to protocol non-adherence (eg, as randomised analysis), and any statistical methods to handle missing data (eg, multiple imputation)
	**Methods: Monitoring**		
**14, Suppl.**	Data monitoring	21a	Composition of data monitoring committee (DMC); summary of its role and reporting structure; statement of whether it is independent from the sponsor and competing interests; and reference to where further details about its charter can be found, if not in the protocol. Alternatively, an explanation of why a DMC is not needed
**14, Suppl.**		21b	Description of any interim analyses and stopping guidelines, including who will have access to these interim results and make the final decision to terminate the trial

**Suppl.**	Harms	22	Plans for collecting, assessing, reporting, and managing solicited and spontaneously reported adverse events and other unintended effects of trial interventions or trial conduct
**Suppl.**	Auditing	23	Frequency and procedures for auditing trial conduct, if any, and whether the process will be independent from investigators and the sponsor
	**Ethics and dissemination**		
**14-15**	Research ethics approval	24	Plans for seeking research ethics committee/institutional review board (REC/IRB) approval
**Suppl.**	Protocol amendments	25	Plans for communicating important protocol modifications (eg, changes to eligibility criteria, outcomes, analyses) to relevant parties (eg, investigators, REC/IRBs, trial participants, trial registries, journals, regulators)
**10,11**	Consent or assent	26a	Who will obtain informed consent or assent from potential trial participants or authorised surrogates, and how (see Item 32)
**N/A**		26b	Additional consent provisions for collection and use of participant data and biological specimens in ancillary studies, if applicable
**14, Suppl.**	Confidentiality	27	How personal information about potential and enrolled participants will be collected, shared, and maintained in order to protect confidentiality before, during, and after the trial
**16**	Declaration of interests	28	Financial and other competing interests for principal investigators for the overall trial and each study site
**Suppl.**	Access to data	29	Statement of who will have access to the final trial dataset, and disclosure of contractual agreements that limit such access for investigators
**Suppl.**	Ancillary and post-trial care	30	Provisions, if any, for ancillary and post-trial care, and for compensation to those who suffer harm from trial participation

For peer review only

**3, Suppl.**	Dissemination policy	31a	Plans for investigators and sponsor to communicate trial results to participants, healthcare professionals, the public, and other relevant groups (eg, via publication, reporting in results databases, or other data sharing arrangements), including any publication restrictions
		31b	Authorship eligibility guidelines and any intended use of professional writers
		31c	Plans, if any, for granting public access to the full protocol, participant-level dataset, and statistical code
	**Appendices**		
**Suppl.**	Informed consent materials	32	Model consent form and other related documentation given to participants and authorised surrogates
**N/A**	Biological specimens	33	Plans for collection, laboratory evaluation, and storage of biological specimens for genetic or molecular analysis in the current trial and for future use in ancillary studies, if applicable

*It is strongly recommended that this checklist be read in conjunction with the SPIRIT 2013 Explanation & Elaboration for important clarification on the items. Amendments to the protocol should be tracked and dated. The SPIRIT checklist is copyrighted by the SPIRIT Group under the Creative Commons "Attribution-NonCommercial-NoDerivs 3.0 Unported" license.

For peer review only
